# Comprehensive Review of Extracellular Vesicles in Thyroid Cancer: From Methodological Approaches to Biological Functions and Clinical Applications

**DOI:** 10.3390/ijms27167337

**Published:** 2026-08-17

**Authors:** Sonja Šelemetjev, Tijana Išić Denčić, Ninoslav Mitić

**Affiliations:** Institute for the Application of Nuclear Energy—INEP, University of Belgrade, 11000 Belgrade, Serbia; sonja@inep.co.rs (S.Š.); tijana@inep.co.rs (T.I.D.)

**Keywords:** EVs, exosomes, biomarkers, non-coding RNA, protein, liquid biopsy, diagnosis, prognosis, prediction, therapy

## Abstract

Thyroid cancer (TC) is one of the most common endocrine malignancies and ranks among the ten most frequently diagnosed cancers worldwide, highlighting the need for improved diagnostic and monitoring strategies. Extracellular vesicles (EVs) are nano-sized, membrane-enclosed particles released by nearly all cell types that carry selectively sorted bioactive cargo capable of influencing the behavior and fate of recipient cells. Although their molecular composition is shaped by their cells of origin, cargo loading is a regulated process that contributes to EV-mediated intercellular communication and cell-specific targeting. In TC, EVs have emerged as important mediators of tumor progression and microenvironment modulation. Recent advances underscore the diagnostic and prognostic value of EVs as non-invasive biomarkers, particularly through the detection of EV-associated non-coding RNAs, proteins, lipids, and other molecular signatures. However, methodological variability and limited clinical validation remain key barriers to clinical translation. Further preclinical and clinical studies are needed to establish the role of EVs in liquid biopsy and personalized targeted therapy for TC. This review provides a comprehensive and systematic overview of EVs in TC, with particular emphasis on methodological factors influencing EV research, including sample-specific isolation and characterization strategies. Based on a systematic literature survey, this review integrates current knowledge on EV-associated cargo, biological functions, biomarker and therapeutic potential while critically evaluating methodological challenges and outlining future directions for clinical translation.

## 1. Introduction

Tumors derived from thyroid epithelial cells are among the most common clinically recognized endocrine neoplasms [1]. They include a spectrum of cytohistomorphologically overlapping lesions with varying tumor biology, ranging from benign tumors via slow-growing carcinomas to rapidly fatal malignant neoplasms [2]. Although most patients with thyroid tumors have a good prognosis, there is a subset of high-risk patients who require a more intensive therapy approach, and the identification of these patients poses difficulties for clinicians [3,4,5,6]. Extracellular vesicles (EVs) have emerged as a promising area of research, offering a minimally invasive way to improve patient management, as the EV cargo can reflect the molecular state of the cells from which they originate or be selectively sorted. In thyroid cancer (TC), EVs carry oncogenic molecules that may contribute to tumor aggressiveness. A better understanding of TC-derived EVs could improve disease surveillance, support treatment monitoring, and help guide future therapeutic strategies.

## 2. Thyroid Cancer

Today, TC ranks among the ten most frequently diagnosed cancers worldwide. Malignant tumors of the thyroid gland originating from follicular epithelium are generally classified as well-differentiated (papillary, follicular, and oncocytic thyroid carcinomas) (DTC), poorly differentiated (PDTC), and undifferentiated (anaplastic) thyroid carcinomas (ATC) [7,8,9]. The biological behavior of these subtypes of TC is highly divergent, as reflected by differences in their patterns of metastases, clinical aggressiveness, and prognosis.

Papillary thyroid carcinoma (PTC) is the most frequent thyroid malignancy, accounting for approximately 80% of all TC. PTC is generally highly curable, with a favorable prognosis for most patients (10-year disease-specific survival rate exceeds 90%). However, PTC has a strong propensity to metastasize to regional lymph nodes, which increases the risk of local-regional relapses and affects the course of the disease. 5–10% of the patients who develop local recurrence and/or distant metastases do not respond to conventional therapies, and consequently, these patients may die from the disease. Follicular thyroid carcinoma (FTC) accounts for 10–15% of all TC. In contrast to PTC, which often spreads to lymph nodes in the neck, FTC commonly spreads hematogenously to bones and lungs. FTC may be more challenging to treat than PTC, and the survival rate of the patients depends on the FTC level of invasiveness. Oncocytic thyroid carcinoma (OTC), previously known as Hürthle cell carcinoma, is a rare subtype of TC (3–5% of all TC). It originates in the malignant transformation of thyrocytes into oncocytic cells. The prognosis of OTC is generally favorable with early diagnosis and treatment. However, this subtype of TC has more aggressive biological behavior than PTC and FTC; it may metastasize via lymph and blood vessels, and it is harder to treat. Consequently, the survival rate of OTC patients declines significantly with advanced disease stage, larger tumor size, or metastasis. In contrast to DTC, ATC is a rare (3–5% of all TC), undifferentiated, highly aggressive TC subtype. It progresses rapidly and frequently metastasizes distantly, resulting in a 1-year survival rate usually below 20% [10]. While precision care and emerging therapies, including timely targeted or multimodal treatments, have led to improved outcomes for select patient groups—showing 2-year survival rates of approximately 24% to 42% and 5-year survival rates around 23% in certain institutional series—the overall prognosis remains poor [11,12,13]. PDTC, accounting for between 3 and 5% of all TC, represents transitional forms between well-differentiated and undifferentiated carcinomas of the thyroid gland. The cells of PDTC have more abnormalities compared to the ones in well-differentiated carcinomas. 

The new classification of TC (The WHO classification of tumors: endocrine and neuroendocrine tumors 5th ed., Vol. 10) provides updated international standards for diagnosing endocrine cancers, focusing on molecular pathology, histopathology, and risk assessment. It reorganizes tumors based on a combination of cellular origin, molecular features, and grade, rather than just morphology [7,14,15,16]. Some of the key changes in the classification of tumors derived from thyrocyte cell transformation include the introduction of high-grade categories for follicular cell-derived carcinomas (PDTC and high-grade differentiated carcinomas are now grouped together), the new status for OTCs, the classification of invasive encapsulated follicular variant of PTC as a distinct malignant neoplasm, and the introduction of the new category of low-risk neoplasms, which includes non-invasive follicular thyroid neoplasm with papillary-like nuclear features (NIFTP), tumors of uncertain malignant potential, and hyalinizing trabecular tumors [16,17,18].

As can be seen, while most thyroid tumors follow an indolent course, important clinical challenges remain beyond conventional prognostic assessment [3,6,19,20]. In daily practice, clinicians must distinguish malignant from benign lesions among indeterminate thyroid nodules, reduce diagnostic uncertainty before surgery, and avoid unnecessary thyroidectomy or overtreatment in patients with low-risk disease. At the same time, more precise tools are needed to identify patients likely to develop aggressive behavior, radioiodine-refractory differentiated TC, or clinically significant recurrence during follow-up. Current imaging, needle-based cytology, molecular testing, and standardized risk-stratification systems have improved TC detection and management, but they do not fully resolve these needs, particularly when available markers are postoperative, tissue-dependent, or insufficiently informative for dynamic surveillance [21,22,23,24]. Although many factors that affect TC prognosis, such as age and gender of the patient, histologic subtype, tumor size, presence of extrathyroidal extension or lymph-node metastases (LNM), and persistence of distant metastases, are known nowadays [3,5,19], many of those factors are not independent predictors of the patient’s outcome. Consequently, there is a strong clinical rationale for minimally invasive biomarkers that can support preoperative decision-making, guide individualized treatment intensity, detect radioiodine-refractory biology earlier, and improve monitoring for recurrent disease. In this context, EV-based biomarkers are of increasing interest because they may reflect tumor-derived molecular information in accessible biofluids and therefore complement existing diagnostic, prognostic, and predictive strategies. These biomarkers could potentially enhance patient outcomes by enabling more tailored therapeutic approaches and timely interventions.

As research in this area progresses, the integration of EV-based diagnostics into clinical practice may revolutionize the management of various malignancies, including TC. Ongoing studies are exploring the specificity and sensitivity of these biomarkers, aiming to establish standardized protocols for their implementation. As evidence accumulates, clinicians may gain valuable tools for early detection and personalized treatment plans, ultimately improving survival rates and quality of life for TC patients.

**Figure 1:**
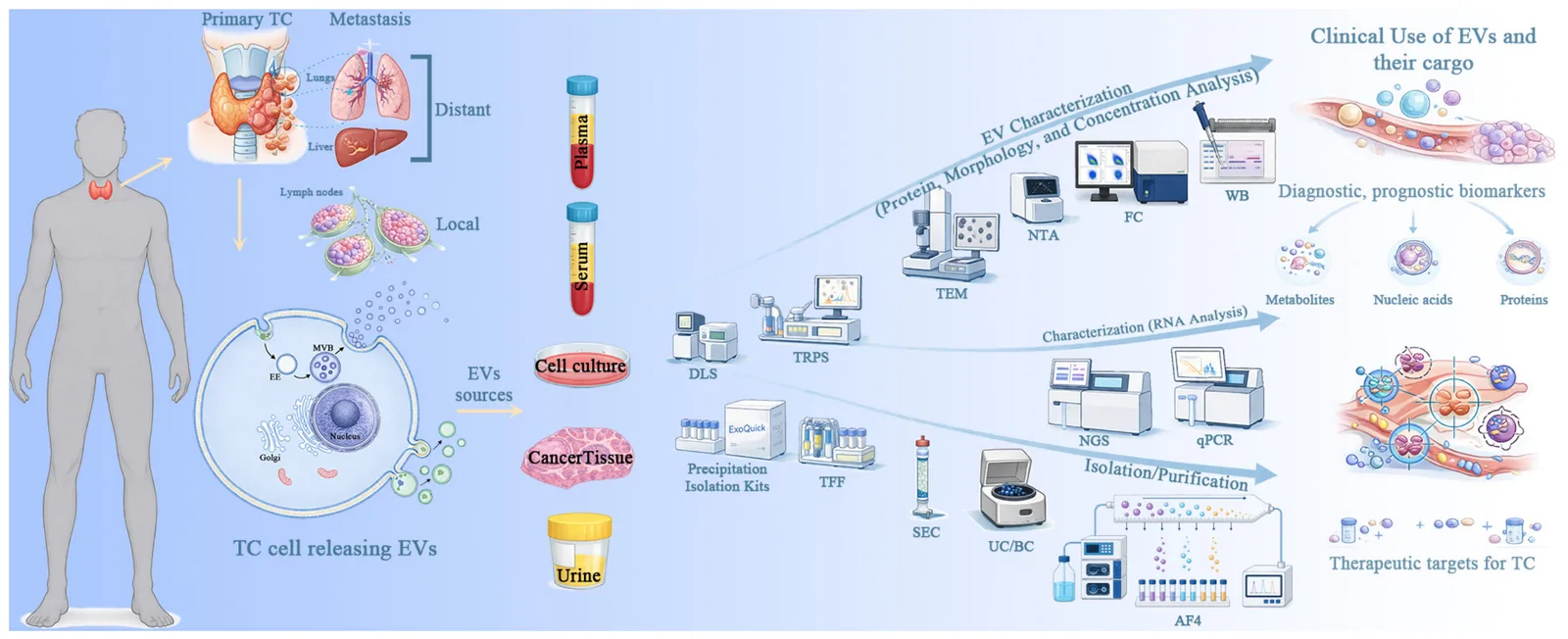
Extracellular vesicles (EVs) in thyroid cancer (TC): from biogenesis and isolation to molecular profiling and clinical applications. AF4, Asymmetric flow field-flow fractionation; BC, benchtop centrifuge; DLS, dynamic light scattering; FC, flow cytometry; NGS, next-generation sequencing; NTA, nanoparticle tracking analysis; qPCR, quantitative polymerase chain reaction; SEC, size-exclusion chromatography; TEM, transmission electron microscopy; TFF, tangential flow filtration; TRPS, tunable resistive pulse sensing; UC, ultracentrifugation; WB, Western blot.

This review provides a comprehensive and systematic overview of currently available knowledge of TC-derived EVs and their molecular cargo. Emphasis is placed on methodological aspects, including EV isolation and characterization approaches, as well as the diversity of biological sample types used for EV analysis (Figure 1). By integrating methodological considerations with biological functions and current diagnostic, prognostic and therapeutic applications, this review offers a unified framework that facilitates comparison between studies, identifies current knowledge gaps, and may support the design of future translational and clinical investigations.

## 3. Extracellular Vesicles

Extracellular vesicles (EVs) are small, heterogeneous, nano-sized particles enclosed by a lipid bilayer [25]. Released by almost all cell types, EVs facilitate intercellular communication by transferring proteins, lipids, glycans, and nucleic acids to recipient cells, thereby modulating their physiological state [26,27]. Once considered cellular debris, they are now recognized as biologically active mediators with growing diagnostic and therapeutic potential, particularly in cancer [28,29,30].

There are several classes of extracellular vesicles (EVs) that differ in their biogenesis, size, and molecular composition [22,31]. The major subtypes include exosomes, microvesicles (also termed ectosomes or shedding vesicles), and apoptotic bodies. Exosomes (approximately 30–150 nm) originate from the endosomal pathway through inward budding of multivesicular bodies followed by fusion with the plasma membrane. Microvesicles (100–1000 nm) are generated by direct outward budding and fission of the plasma membrane, whereas apoptotic bodies (500–2000 nm) are released during programmed cell death and contain fragmented cellular components [22,32].

The extracellular secretome has recently expanded beyond these classical EV categories. Migrasomes (500–3000 nm) are produced during migracytosis and participate in intercellular communication associated with cell migration, whereas exophers (3.5–4 μm), a recently described class of extracellular particles, are thought to mediate the extrusion of damaged organelles and protein aggregates [33,34,35]. Large oncosomes (1–10 μm), shed predominantly by highly migratory tumor cells, are enriched in oncogenic proteins and nucleic acids and contribute to tumor progression and microenvironment remodeling [36,37,38]. In parallel, several non-membranous extracellular nanoparticles have been identified, including lipoproteins, exomeres (~35 nm), and supermeres (~30 nm), which carry distinct repertoires of proteins, RNAs, and metabolites associated with cancer and other diseases [39,40,41,42]. Because these particles frequently co-isolate with EVs, particularly following differential ultracentrifugation (DUC), they contribute to heterogeneous extracellular particle populations, and their distinction from membrane-enclosed EVs is only beginning to emerge and has not yet been universally accepted [43].

To improve consistency across the field, the International Society for Extracellular Vesicles (ISEV) introduced the Minimal Information for Studies of Extracellular Vesicles (MISEV) guidelines, updated in 2014, 2018, and 2023, which standardize EV terminology, experimental design, and reporting practices [25,31,44,45]. Despite these recommendations, the term exosome continues to be used frequently throughout the literature without experimental evidence of an endosomal origin. Because unequivocally demonstrating EV biogenesis remains challenging, the MISEV guidelines recommend using the generic term extracellular vesicles, unless endosomal origin has been established. Accordingly, EVs should be classified using operational criteria based on measurable properties, including size, density, molecular composition, or cell of origin. Throughout this review, we follow these recommendations by using the generic term EVs and, where appropriate, the operational size-based categories small EVs (<200 nm, sEVs) and large EVs (>200 nm, lEVs) [25].

### 3.1. Isolation of TC-Derived EVs

Numerous EV isolation methods have been developed, each exploiting different physicochemical properties, such as size, density, surface charge, or surface marker expression (Table 1, Figure 1). Because no single approach simultaneously achieves high recovery, purity, scalability, and preservation of EV integrity, method selection depends on both the biological sample and the intended downstream application. Consequently, hybrid workflows combining complementary approaches are increasingly recommended to improve EV purity while maintaining recovery, consistent with recent MISEV guidelines [25,31]. 

DUC remains the most widely used approach because of its simplicity and broad applicability, although it frequently co-isolates proteins and lipoproteins [46]. Density-gradient ultracentrifugation (DGUC) and size -exclusion chromatography (SEC) generally provide higher-purity preparations, while ion-exchange chromatography (IEC) offers an alternative chromatographic strategy by exploiting the net negative surface charge of EVs for selective enrichment [47,48,49]. Polymer precipitation (PEG) provides a rapid and inexpensive alternative at the expense of specificity [50,51]. Filtration and tangential flow filtration (TFF) facilitate scalable processing of large sample volumes, whereas immunoaffinity capture enables enrichment of disease-specific EV populations [46,52,53,54]. Emerging technologies—including microfluidics, AF4, acoustic separation, and other label-free platforms—offer increasing automation and analytical sensitivity but still require further clinical validation [39,55,56,57,58,59,60].

In TC research, however, the choice of isolation strategy is particularly important because half of the surveyed studies analyzed plasma or serum (including studies with multiple biological sources, Supplementary Table S1), where tumor-derived EVs constitute only a small fraction of the total circulating EV population. Most circulating EVs originate from hematopoietic cells, particularly platelets, increasing background contamination and reducing the sensitivity for detecting tumor-derived biomarkers [61,62,63]. Consistent with this challenge, our literature survey revealed that ultracentrifugation and polymer-based precipitation kits (primarily ExoQuick) remain the predominant isolation methods in TC studies (Supplementary Table S1), whereas relatively few studies employ higher-purity workflows such as SEC, DGUC, affinity capture, or combined isolation strategies recommended by the MISEV guidelines. Additionally, we observed inconsistencies in the nomenclature used for commercial precipitation-based EV isolation reagents, with products occasionally reported under generic or variable names and without manufacturer or product identifiers. Such incomplete reporting complicates comparisons between studies and further limits methodological standardization and reproducibility (Supplementary Table S1). 

**Table 1:** Overview of extracellular vesicle (EV) isolation methods.

Isolation Method	Separation Principle	Main Advantages	Main Limitations	CS *	Samples	Best Suited for ***	Reference(s)
Differential ultracentrifugation (DUC)/benchtop centrifugation (BC)	Sequential centrifugation according to sedimentation rate	Widely established (DUC); reagent-free; inexpensive; compatible with many sample types; relatively fast (BC)	Time-consuming and requires an ultracentrifuge (DUC); co-isolation of contaminants; possible EV aggregation and deformation; limited scalability	★★☆	Cell culture medium, plasma, serum	Exploratory research; in vitro functional studies; initial EV isolation from cell culture	[46,64]
Density-gradient ultracentrifugation (DGUC)	Separation according to buoyant density in sucrose or iodixanol gradients	Highest purity; excellent removal of protein and lipoprotein contaminants	Labor-intensive; low throughput; prolonged processing	★☆☆	Cell culture medium, plasma, serum, urine	Mechanistic studies; proteomics; RNA sequencing; high-purity EV characterization	[52]
Polymer precipitation (PEG)	Reduced EV solubility induces vesicle precipitation	Rapid; inexpensive; no specialized equipment; suitable for large sample volumes	Low specificity; co-precipitation of proteins and lipoproteins; often requires additional purification	★★☆	Cell culture medium, plasma, serum, urine	High-throughput screening; pilot biomarker studies; clinical cohorts where maximum recovery is prioritized over purity	[50,51]
Size -exclusion chromatography (SEC)	Separation according to size through porous beads	High purity; preserves EV integrity and biological activity; highly reproducible	Sample dilution; lower recovery from small volumes	★★★	Cell culture medium, plasma, serum, urine	Biomarker discovery; proteomic and transcriptomic profiling; functional studies requiring high EV purity; plasma- and serum-derived EVs purification	[47,48]
Ion -exchange chromatography (IEC)	Separation according to EV surface charge	Mild isolation conditions; scalable; high recovery; compatible with clinical samples	Less extensively validated; performance depends on EV surface charge variability	★★☆	Cell culture medium, plasma, serum, urine	Plasma- and serum-derived EVs purification; complementary chromatographic purification; translational biomarker research	[49]
Ultrafiltration (UF)	Separation by membrane filtration according to pore size	Fast; inexpensive; concentrates EVs efficiently	Membrane clogging; possible vesicle deformation; co-retention of soluble contaminants	★★☆	Cell culture medium, plasma, serum, urine	EV concentration before downstream purification; processing of low-concentration samples	[46]
Tangential flow filtration (TFF) **	Crossflow membrane filtration minimizes fouling while concentrating EVs	Highly scalable; excellent recovery; preserves EV integrity; GMP-compatible	Usually requires SEC or DGUC polishing for optimal purity	★★★	Cell culture medium, plasma, serum, urine	Large-volume clinical samples; biobanking; GMP-compatible EV production; preclinical and therapeutic EV manufacturing	[53]
Affinity capture	Capture of EVs expressing specific surface molecules using divergent affinity ligands	Highest specificity; isolation of disease- or tissue-specific EV populations	Marker-dependent selection bias; relatively low yield; higher cost	★★★	Cell culture medium, plasma, serum, urine	Isolation of tissue- or disease-specific EVs; precision biomarker studies; liquid biopsy	[52,54]
Microfluidics	Miniaturized platforms integrating size-, affinity-, acoustic-, or flow-based separation	Rapid; high sensitivity; automated; needs minimal sample volume; point-of-care potential	Device standardization and scalability remain challenging	★★★	Cell culture medium, plasma, serum, urine, FNA	Point-of-care diagnostics; liquid biopsy; automated clinical workflows; low-volume clinical samples	[60]
Acoustic wave separation	Standing or traveling acoustic waves separate EVs according to size and density	Label-free; gentle; preserves EV integrity; minimal contamination	Specialized instrumentation; limited widespread implementation	★★☆	Whole blood, plasma, serum	Rapid label-free isolation from blood; clinical diagnostic workflows (emerging)	[57]
Asymmetric flow field-flow fractionation (AF4)	Hydrodynamic separation according to diffusion coefficient and particle size	Exceptional resolution of EV subpopulations and extracellular nanoparticles	Expensive instrumentation; relatively low throughput	★☆☆	Cell culture medium, plasma, serum	Separation of EV subtypes; advanced research applications; EV subtype characterization ****	[39]

* Clinical suitability (CS): ★☆☆ = primarily research use; ★★☆ = translational/limited clinical implementation; ★★★ = high clinical and liquid biopsy potential. ** Large-volume samples. *** Recommended applications reflect the current literature and the authors’ perspective regarding the most appropriate use of each isolation strategy in extracellular vesicle research, particularly in thyroid cancer. **** When coupled with detectors such as multi-angle light scattering (MALS), dynamic light scattering (DLS), etc. Fine-needle aspiration—FNA.

Considering the strengths and limitations of currently available methods used in TC studies (Table 1, Supplementary Table S1), combined isolation workflows are likely to provide the most reliable strategy for biomarker discovery, whereas scalable approaches such as TFF or microfluidic platforms may be better suited for future clinical implementation. Conversely, affinity-based isolation using thyroid-specific markers may facilitate enrichment of tumor-derived EVs and improve analytical specificity for liquid biopsy applications. 

Furthermore, inconsistent EV dosing in functional studies and the frequent presence of co-isolated non-vesicular components remain important sources of variability that should be considered when interpreting biological findings and assessing candidate biomarkers in TC.

### 3.2. Molecular Characterization of TC EVs

Following isolation, EVs require comprehensive characterization to verify their identity, assess sample purity, and define their molecular cargo, ensuring that downstream analyses reflect biologically relevant vesicle populations. According to the MISEV2018 and MISEV2023 guidelines, characterization should include representative markers of membrane-associated proteins, cytosolic proteins, and potential non-EV contaminants, particularly when isolation protocols or pre-analytical conditions differ [25,31]. The updated MISEV2023 guidelines further recommend demonstrating enrichment or depletion of these markers relative to the source material to strengthen evidence for EV identity and purity. sEVs are commonly enriched in tetraspanins (CD9, CD63, CD81), ESCRT-associated proteins (TSG101, Alix), and heat shock proteins, whereas larger plasma membrane-derived EVs are relatively enriched in integrins, selectins, cytoskeletal proteins, and membranes containing phosphatidylserine and cholesterol [65,66,67]. However, because no single molecular marker uniquely defines a specific EV subtype, reliable characterization requires the complementary use of multiple analytical approaches [25,31]. EV characterization generally combines measurements of particle size and concentration, morphological assessment, and analysis of EV-associated proteins and nucleic acids (Figure 1). Standardization of these complementary techniques is essential to ensure reproducibility and enable meaningful comparisons across studies. Table 2 summarizes the principal EV characterization methods applied in TC research, together with their main advantages and limitations. 

Based on our literature survey, most TC studies rely on a combination of TEM/SEM, NTA, and Western blotting (Supplementary Table S1), whereas advanced single-particle and high-resolution analytical techniques remain rarely employed. Consequently, broader implementation of complementary characterization approaches is likely to improve the robustness, reproducibility, and comparability of TC EV studies.

### 3.3. Molecular Cargo of TC-Derived EVs

EV molecular cargo mediates intercellular communication between parental cells and the microenvironment. It may be selectively sorted or reflect the molecular state of the cells of origin. The cargo comprises biomolecules within the vesicle lumen and on the outer membrane. 

In addition to proteins and lipids, TC EVs transport diverse nucleic acids, including messenger RNAs (mRNAs), microRNAs (miRNAs), long non-coding RNAs (lncRNAs), circular RNAs (cir cRNAs), transfer RNA fragments (tRFs), Y RNAs, small nucleolar RNAs (snoRNAs), and DNA, which collectively regulate gene expression and influence tumor growth, invasion, metastasis, immune modulation, and therapeutic response [68,69,70,71,72,73,74,75,76,77,78,79,80,81,82,83]. Owing to their remarkable stability in biological fluids and protection from enzymatic degradation, EV-associated biomolecules have emerged as promising candidates for liquid biopsy, disease monitoring, prognostic assessment, prediction of therapeutic response, and precision oncology [84].

The analysis of published TC EV studies (Supplementary Table S1) revealed that investigations have predominantly focused on miRNA (~49%) and protein cargo (~41%), whereas lncRNAs (~7%), circRNAs (~4%), and other non-coding RNAs or EV-associated biomolecules have received considerably less attention. Furthermore, only approximately 12% of studies simultaneously investigated multiple EV cargo classes, indicating that comprehensive integrated molecular profiling approaches of TC-derived EVs remain largely underexplored. Among the investigated TC biomarkers, EV-associated miRNAs currently represent the most extensively studied biomarker class, with the strongest evidence supporting their clinical utility. In particular, miR-146b-5p, miR-21-5p, members of the miR-221/222 family, and several recently proposed multi-miRNA signatures have consistently shown promising diagnostic and prognostic performance across independent studies. In comparison, EV-associated proteins, including programmed death-ligand 1 (PD-L1), thyroglobulin (Tg), and galectin-3, have shown encouraging but comparatively less well-validated clinical potential [74,76,77,78,79,80,81,82,83,84,85,86,87,88,89,90,91,92,93,94,95,96,97,98,99,100,101,102,103,104,105,106]. Despite the well-established importance of aberrant glycosylation in thyroid cancer biology, studies investigating EV glycosylation or glycan-based biomarkers remain remarkably scarce, highlighting an important and largely unexplored area for future biomarker discovery. Nevertheless, most proposed EV biomarkers have been identified in relatively small TC patient cohorts using heterogeneous isolation and characterization workflows, limiting cross-study comparability and clinical validation. Future studies integrating complementary EV cargo classes together with standardized methodologies are therefore expected to improve biomarker robustness, patient stratification, and the clinical translation of TC-derived EVs.

## 4. The Role of EVs in the Pathogenesis of TC

**Figure 2:**
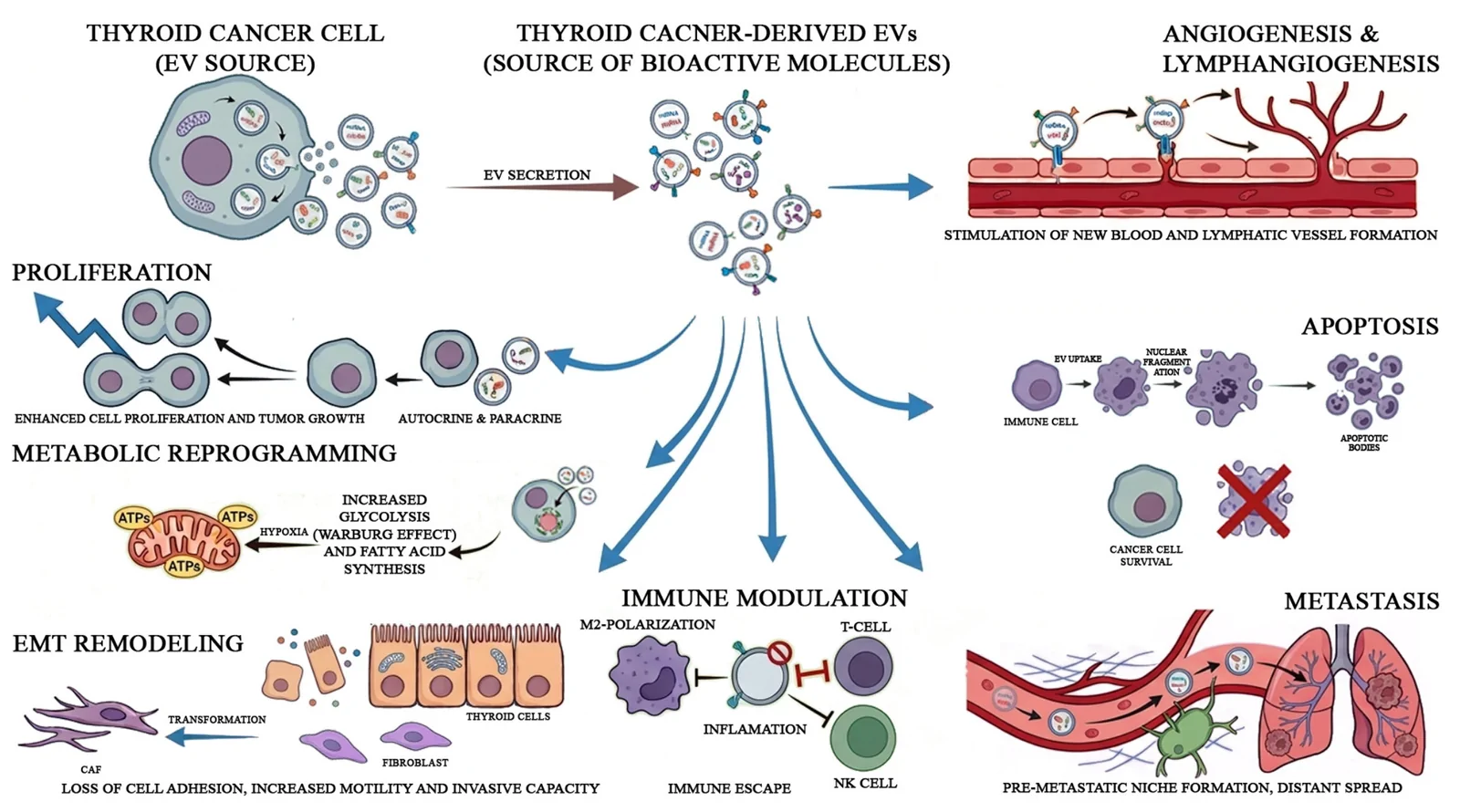
Schematic illustration of the role of extracellular vesicles (EVs) in thyroid cancer pathogenesis. ATP, adenosine triphosphate; CAF, cancer-associated fibroblast; EMT, epithelial–mesenchymal transition; EV, extracellular vesicles; NK cell, natural killer cell.

The biological functions of EVs are largely determined by their molecular cargo. The selective packaging of molecular constituents into EVs, followed by their subsequent uptake by recipient cells, profoundly influences various cellular processes within the tumor microenvironment and at distant metastatic sites (Figure 2). Recent research highlights how these nano-sized biological units facilitate TC progression by horizontally transferring bioactive cargo to both neighboring and distant recipient cells [71,107]. Interactions between TC cells and stromal cells alter the contexture of released EVs, influencing tumor progression, metastasis, and immune interactions, with varying expression levels across different cancer stages, suggesting their potential as diagnostic and prognostic biomarkers, as well as therapeutic targets [107,108,109].

### 4.1. EV-Mediated TC Proliferation and Tumor Growth

Tumor-derived EVs significantly contribute to the progression of cancer by selectively transporting oncogenic cargo that reshapes the tumor microenvironment [69,109]. These vesicles actively promote the proliferation of surrounding tumors and stromal cells through the transfer of growth factors and oncogenic signaling molecules, thereby accelerating overall tumor expansion (Figure 2) [107].

Early investigations identified several lncRNAs, including CDKN2B AS1 and DOCK9 AS2, as key mediators of TC cell proliferation and invasion [99]. CDKN2B AS1 has been specifically associated with TC stem cell–mediated tumorigenesis—it functions by sponging miR-122-5p, resulting in the upregulation of Prolyl 4-Hydroxylase Subunit Alpha 1 (P4HA1), promoting viability, stemness, migration and invasion of TC cells in vitro. Animal-model data further suggest that modulation of this axis can increase tumor volume and weight in vivo [98]. These findings provide coherent biological support for CDKN2B-AS1 as an oncogenic EV cargo; however, the translational relevance is limited by reliance on selected cell lines, engineered gain- or loss-of-function models, and xenograft systems that do not fully reproduce the immune, stromal, and endocrine context of human TC.

At the clinical level, the strongest evidence currently relates to the biomarker potential of EV-associated miRNAs rather than to direct proof of causality. MiR-146b-5p and miR-222-3p were recognized as predominant EV markers in PTC [94,110]. The functional assays in PTC cell lines indicate that miR-146b-5p and miR-222-3p can enhance migration and invasion, partly through modulation of tumor-suppressive pathways such as Sma and Mad Related Proteins Family Member 4 (SMAD4) and activation of the Ak Strain Transforming (AKT)-related signaling [94,110].

Similarly, EV-associated proteins such as Annexin A1 (ANXA1) provide an example of a biologically plausible but still largely preclinical mechanism. Elevated levels of ANXA1 derived from EVs produced by ATC cell line SW579 were shown to promote the proliferation and invasion of thyroid epithelial cells in vitro, contributing to their malignant transformation [111]. Xenograft experiments further indicate that SW579-derived EVs ANXA1 can enhance tumor growth in vivo [112]. Nevertheless, these data should not be overgeneralized to clinical ATC biology without caution, because they are based mainly on experimental models and do not yet demonstrate that ANXA1-positive EVs predict progression, therapeutic response, or survival in well-characterized patient cohorts.

### 4.2. EV-Induced TC Metabolic Reprogramming

A hallmark of TC progression is EV-induced metabolic reprogramming [109]. At present, the strongest support for EV-mediated metabolic effects is also preclinical, particularly from TC cell-line experiments and, in selected studies, xenograft animal models. Therefore, mechanistic statements regarding EV-induced glycolysis, lipid remodeling, or hypoxia adaptation should be framed as biologically plausible and experimentally supported, but not yet clinically established.

In vitro evidence suggests that EVs released by TC cells can transfer lipid mediators, including cholesterol and phospholipids, and may thereby support tumor-cell survival under nutrient-deprived or otherwise stressful conditions [109]. By transferring these lipids and other molecules, their cargo helps TC cells shift their metabolism (e.g., increasing glycolysis and fatty acid synthesis) to generate energy and precursors needed for survival and proliferation under stress. However, this conclusion is mainly derived from controlled experimental systems; therefore, EV-associated metabolic reprogramming remains insufficiently defined in human TC. Functional assays further showed that circular RNA Phosphoglycerate Dehydrogenase (CircPHGDH) is a key driver that promotes TC growth by up-regulating Pyruvate Kinase Muscle Isoform 2 (PKM2), a critical glycolytic enzyme that shifts cellular metabolism toward aerobic glycolysis (the Warburg effect) [113]. Additionally, circular RNA Coiled-Coil Domain Containing 66 (circCCDC66) facilitates rapid proliferation by targeting metabolic regulators, such as pyruvate dehydrogenase kinase 4 (PDK4) and AKT, via sponging miR-211-5p, leading to increased expression of PDK4 and further enhancing glucose metabolism [111]. In 2023, Powell et al. established that miR-210-3p levels were elevated in hypoxic settings inside the ATC cell line SW1736 [97]. MiR-210-3p was secreted in EVs with Argonaute 2 (AGO2), suggesting its viability as a biomarker for cellular and extracellular hypoxia. Additional preclinical work in PTC indicates that hypoxia-derived EVs may enrich miR-221-3p and promote proliferation, migration, and invasion through targeting Zinc Finger AN1-Type Domain-Containing Protein 5 (ZFAND5) [114]. The suppression of miR-221-3p diminished tumor proliferation in PTC models, underscoring its potential as a biomarker for diagnosis and prognosis, or eventual target for therapeutic intervention. Nevertheless, these findings remain primarily mechanistic and model-based; they require validation in TC patient-derived samples, longitudinal cohorts, and clinically annotated datasets before miR-210-3p or miR-221-3p can be considered reliable markers of hypoxia-driven progression or therapeutic resistance.

The clinical argument for applying EV-based metabolic markers is attractive but remains preliminary. The studies support a plausible role for non-coding RNA networks in metabolic reprogramming, yet they do not by themselves demonstrate that EV-packaged molecules are clinically actionable biomarkers or may be therapeutic targets. Therefore, EV-associated metabolic reprogramming could be regarded as a promising research area with translational potential, but routine clinical application requires standardized methodology, independent validation, prospective study design, and demonstration of added value over current diagnostic and prognostic tools.

### 4.3. TC-Derived EVs in Promoting Epithelial–Mesenchymal Transition and Extracellular Matrix Remodeling

EMT represents a fundamental cellular reprogramming process in which epithelial cells progressively lose their polarity and intercellular adhesion, acquiring mesenchymal traits such as enhanced motility and invasive capacity. EVs play a role in orchestrating this transition by serving as carriers of regulatory molecules that mediate communication between malignant TC cells and the surrounding stromal cells. EV-mediated crosstalk is particularly evident in the dynamic interaction between thyroid tumor cells and resident fibroblasts. In vitro studies showed that TC EVs can reprogram quiescent fibroblasts into cancer-associated fibroblasts (CAFs), a highly active stromal population that further amplifies tumor aggressiveness [69,115]. Once activated, CAFs reciprocally secrete EVs enriched in pro-invasive and pro-remodeling factors, reinforcing a bidirectional communication loop that sustains EMT, extracellular matrix (ECM) degradation, and tumor expansion. Fibroblasts within the tumoral microenvironment internalize substantially more EVs than fibroblasts in non-tumoral tissue, enabling a more efficient and persistent pro-metastatic signaling network. This preferential uptake accelerates ECM remodeling by increasing the abundance of key structural and enzymatic proteins within CAFs [69,115]. As a result, the tumor microenvironment becomes progressively more permissive to TC cell dissemination. As far as that goes, TC EVs are evidently increasingly implicated in EMT and ECM remodeling, the level of evidence differs considerably across studies, and the most direct mechanistic evidence is still preclinical.

In vitro co-culture studies of thyroid tumor cells and fibroblasts have shown that EVs released within tumor–stromal interactions can carry CD147 (also known as EMMPRIN) and are associated with increased secretion or activation of MMP2 and MMP9, enzymes capable of degrading basement-membrane components such as type IV collagen [69,115,116]. This allows thyroid tumor cells to infiltrate surrounding tissues and enter the bloodstream [69,115,117]. However, the findings should be interpreted primarily as cell-culture evidence, because they do not by themselves establish that EV-bound CD147 or EV-associated MMP activity is an independent driver of metastasis in patients. Clinical evidence linking matrix-remodeling mediators to aggressive TC is suggestive but largely associative. The study of Zhang et al., from 2019., showed that serum levels of MMP2, MMP9, Tissue Inhibitor of Metalloproteinases 1 and 2 (TIMP-1, and TIMP-2) declined only in low-risk DTC patients after the surgery [118], while high levels of these enzymes in the peripheral blood or tumor tissue of patients with DTC persisted in patients with aggressive forms of DTC, with LNM, and poorer prognosis [117,118]. These observations support the relevance of ECM-remodeling pathways in disease aggressiveness, but they do not prove that the measured circulating enzymes are EV-derived or that EV-mediated MMP transfer is causally responsible for clinical progression. 

Evidence for EV-associated miRNAs and circRNA networks in EMT is mechanistically compelling but remains mostly based on in vitro functional assays. One of the primary studies identified miR-423-5p as a key regulator of EMT in patients with PTC [119]. Serum-derived EVs from PTC patients exhibited significantly elevated levels of miR-423-5p, and functional experiments demonstrated that its silencing resulted not only in a marked reduction in EV secretion but also in a substantial impairment of tumor cell migration [119]. These patient-derived findings provide a clinically relevant association, whereas the functional role of miR-423-5p is supported mainly by cell-line experiments. A possible mechanism of action was later suggested in the work of Wen et al., who found that circ0039411 acts as a molecular sponge that sequesters miR-423-5p in PTC [120], consequently preventing the degradation of SRY-Box Transcription Factor 4 (SOX4) mRNA and leading to an upregulation of SOX4 protein levels. Increased SOX4 activity enhances aerobic glycolysis (the Warburg effect) and promotes the proliferation and migration of TC cells. Importantly, SOX4 acts upstream of the Wingless-related integration site/beta-catenin (Wnt/β-catenin) signaling pathway, a central regulator of EMT. By facilitating β-catenin activation and nuclear translocation, the miR-423-5p/SOX4 axis induces a transcriptional program characterized by the suppression of epithelial markers (notably E-cadherin) and the upregulation of mesenchymal markers such as vimentin. Reduced E-cadherin expression weakens cell–cell adhesion and disrupts epithelial integrity, while increased vimentin expression enhances cytoskeletal plasticity, enabling tumor cells to adopt a more migratory and invasive phenotype. Collectively, these findings position EV-associated miR-423-5p as a central contributor to TC aggressiveness by integrating metabolic reprogramming, EMT activation, and microenvironmental communication. Its regulatory network—encompassing circRNA sponging, SOX4 stabilization, and Wnt/β-catenin pathway activation—illustrates the complexity of EV-mediated molecular signaling. This model integrates EMT activation with metabolic and migratory phenotypes, but it should be framed as a preclinical regulatory pathway until validated in independent patient cohorts with EV-specific measurements and clinical endpoints.

In a related study, circ007293 was identified as a significant promoter of EMT and cellular invasion in PTC through the miR-653-5p/Paired Box 6 (PAX6) regulatory axis [101]. This work demonstrated that circ007293 functions as a molecular sponge for miR-653-5p, thereby diminishing its inhibitory effect on its downstream target, PAX6. The consequent upregulation of PAX6 enhances EMT-associated signaling pathways, ultimately increasing the invasive and migratory capacities of TC cells. However, this conclusion is principally derived from experimental manipulation in cellular models. At present, there is insufficient clinical validation to determine whether EV-associated circ007293 improves diagnostic discrimination, predicts metastatic risk, or identifies patients who would benefit from targeted therapeutic intervention.

Cancer stem cell-like cells (CSCs)-derived EVs add another layer of biological plausibility, but again the evidence is mostly preclinical. They transfer lncRNAs such as DOCK9-AS2 and Metasis-Associated Lung Adenocarcinoma Transcript 1 (MALAT1) to recipient thyroid cells, thereby intensifying EMT and enhancing malignant phenotypes through activation of the Wnt/β-catenin and Transforming Growth Factor-Beta 1/SMAD Member 2 and 3 (TGF β1/Smad2/3) signaling pathways [99]. Beyond reinforcing the invasive characteristics of malignant cells, CSC-derived EVs also deliver lncRNAs, including MALAT1 and Long Intergenic Non-Protein Coding RNA, Regulator of Reprogramming (linc-ROR), to non-cancerous thyroid cells, effectively inducing EMT and reprogramming the surrounding microenvironment to support thyroid tumor growth and progression. These studies support the concept that CSC-derived EVs may help disseminate malignant traits; however, they rely heavily on selected cell models and engineered experimental systems, and their relevance to spontaneous human tumor evolution remains to be established. In contrast, proteomic assays provided more clinical evidence. Namely, significantly elevated levels of RAS Oncogene Family Member 21 (RAB21) were noted in EVs isolated from FTC patients compared with serum-derived EVs in follicular thyroid adenoma (FTA) patients [121]. This finding highlights the potential of specific EV-associated proteins as biomarkers to discriminate malignant from benign follicular thyroid lesions, but its clinical utility requires independent prospective validation. 

Beyond their biochemical effects, in vitro studies showed EV-mediated EMT induction also influences cytoskeletal architecture, cell–cell junction stability, and the expression of EMT-related transcription factors such as Snail Family Transcriptional Repressor 1 (SNAIL), Snail Family Transcriptional Repressor 2 (SLUG), and Zinc Finger E-Box Binding Homeobox 1 and 2 (ZEB1/2) [122,123,124]. Through these combined mechanisms, EVs act not merely as passive carriers but as active drivers of phenotypic plasticity and metastatic competence in TC. 

From a clinical perspective, current clinical investigations provide associations between EV cargo or ECM-remodeling markers and aggressive disease features, but they rarely establish causality, EV specificity, or added value beyond established clinicopathological risk factors. EV-based EMT and ECM-remodeling marker profiling could provide minimally invasive information about tumor–stromal crosstalk, invasive potential, metastatic risk, or malignant transformation, and may complement imaging, cytology, molecular testing, and postoperative surveillance. On the other hand, the assays are not standardized, clinically validated thresholds are lacking, most evidence is preclinical or retrospective, and there is no proof yet that EV-guided decisions improve patient outcomes. Therapeutically, targeting EV release, uptake, or cargo is attractive in principle but remains speculative, with unresolved concerns about specificity, delivery, off-target effects, and disruption of physiological EV communication. Therefore, EV-mediated EMT and ECM remodeling should currently be regarded as a promising research framework and candidate biomarker field, not as an established basis for routine clinical management.

### 4.4. TC-Derived EVs in Angiogenesis and Lymphangiogenesis

EVs promote the formation of new blood vessels (angiogenesis) and lymphatic vessels (lymphangiogenesis) by delivering pro-angiogenic factors and cytokines to endothelial cells, thereby ensuring nutrient supply and facilitating metastatic spread. While there are many studies showing that EV-mediated transfer of specific ncRNAs can reprogram endothelial cells, enhancing their migratory and invasive capabilities, which are crucial for establishing a supportive vascular network within the tumor [125], in TC research, most mechanistic data come from in vitro studies using endothelial-cell models and from selected animal models. Therefore, direct clinical evidence that EVs independently drive angiogenesis or lymphangiogenesis in TC patients remains limited. 

In vitro, hypoxic PTC cells were shown to release EVs enriched in miR-181a, which were taken up by human umbilical vein endothelial cells (HUVECs) and promoted endothelial proliferation and capillary-like network formation [90]. Within recipient cells, miR-181a directly targets and inhibits MLL3 (Mixed-Lineage Leukemia 3), which leads to a reduction in Histone H3 Lysine 4 monomethylation (H3K4me1) levels at the enhancer region of DACT2 (Disheveled-binding Antagonist of β-catenin), causing its downregulation. The suppression of DACT2 activates the YAP signaling pathway, which in turn upregulates VEGF (Vascular Endothelial Growth Factor) transcription, driving tumor angiogenesis and tumor growth in vivo. Animal-model evidence further supports biological relevance: hypoxic PTC-derived EVs enhanced angiogenesis and tumor growth in vivo, and these effects were attenuated by inhibition of miR-181a [87]. Although this provides coherent preclinical evidence for an EV–miR-181a–MLL3/DACT2–YAP–VEGF axis, the evidence remains largely preclinical as it does not yet establish that circulating EV-miR-181a predicts angiogenesis, LNM, treatment response, or survival in independent patient cohorts, nor does it prove that blocking this pathway would be safe or effective in humans. It was further found that FGD5-AS1 isolated from TC EVs promotes angiogenesis and vascular permeability by altering the miR-6838-5p/Vav Guanine Nucleotide Exchange Factor (VAV2) axis in endothelial cells, suggesting a new target for diagnosis and treatment [97]. Similarly, hypoxic PTC-derived EVs enriched in miR-21-5p were shown to suppress TGFBI (Transforming Growth Factor Beta Induced) and COL4A1 (Collagen Type IV Alpha 1 Chain) expression in endothelial cells, thereby increasing endothelial migration and tube formation [109]. Lower levels of TGFBI and COL4A1 proteins lead to increased endothelial cell tube formation and migration, promoting angiogenesis and enhancing the tumor’s blood supply to fuel tumor growth and metastasis. This mechanism was confirmed both in vitro and in nude mouse models, establishing a direct link between hypoxia-induced EV secretion and the nutrient supply necessary for sustained thyroid tumor cell proliferation. However, both mechanisms require further validation in patient-derived EV samples, subtype-specific cohorts, and clinically annotated datasets. 

The clinical rationale for EV-based angiogenesis markers is nevertheless attractive, but there are still many limitations. Most studies rely on HUVEC-based assays, which model vascular endothelial behavior but do not fully recapitulate thyroid tumor vasculature or lymphatic endothelium. On the other hand, animal models, including nude-mouse xenografts, provide useful in vivo support but lack the complete immune system, stromal, endocrine, and vascular context of human TC. Furthermore, many experiments use hypoxia exposure, EV dosing, or cargo manipulation under conditions that may not reflect physiological EV concentrations in patients. Finally, therapeutic targeting of EV release, uptake, or cargo may affect physiological vascular repair and intercellular communication. Therefore, TC-derived EVs should currently be viewed as promising mechanistic and biomarker candidates in angiogenesis research, but not as established diagnostic, prognostic, or therapeutic tools. Future studies should prioritize standardized EV workflows, inclusion of lymphatic endothelial models, patient-derived EV validation, prospective cohorts, and demonstration of added value over existing clinical risk stratification. 

### 4.5. TC-Derived EVs and Immune Modulation

Immune modulation facilitates a more permissive environment for tumor growth and dissemination. TC-derived EVs are plausible mediators of immune modulation, but the strength of evidence varies substantially across immune mechanisms and study designs. Therefore, EVs should be described as candidate contributors to immune escape rather than as fully established drivers of immunosuppression in patients with TC. Current evidence is strongest for the concept that EV cargo can influence T-cell function, macrophage polarization, inflammatory signaling, and immune-checkpoint pathways, but many mechanistic conclusions still rely predominantly on preclinical models [68,109,126,127]. 

In vitro studies indicate EVs released by TC cells can modulate immune responses within the tumor microenvironment by delivering immunosuppressive molecules, such as PD-L1, galectins or High Mobility Group Box 1 (HMGB1), to immune cells, thereby promoting immune evasion and tumor progression [109,127,128]. Mechanistically, HMGB1 activates the Mechanistic Target of Rapamycin Complex 2-AKT- Cellular Myelocytomatosis Oncogene (mTORC2–AKT–c-MYC) signaling pathway, which further enhances the production of PD-L1–positive EVs. In addition, PD-L1 displayed on the surface of TC-derived EVs binds directly to PD-1 receptors on T cells, promoting immunosuppression and reducing the effectiveness of anti-PD-L1 therapies. EV-delivered PD-L1 and suppressive cytokines collectively block the activation of CD8+ T cells. Concurrently, they downregulate activating receptors (such as NKG2D) on Natural Killer (NK) cells, completely impairing the host’s synthesis of crucial effector cytokines like interferon-gamma (IFN-γ) [129,130]. However, the extent to which this mechanism explains resistance to anti-PD-1/PD-L1 therapy in human TC is not yet established, unless supported by patient-derived functional assays or clinical outcome data.

EVs may also connect TC progression with chronic inflammation, but this link is currently supported more by biological plausibility and experimental evidence than by definitive clinical proof [131]. Beyond cancer-specific contexts, EVs have been shown to function as carriers of bioactive molecules capable of modulating cytokine production, inflammatory signaling, and apoptotic pathways in non-tumor disease models, highlighting their broader regulatory potential in immune and tissue responses [132]. However, whether similar EV-mediated mechanisms directly contribute to inflammatory regulation within the TC microenvironment remains insufficiently characterized. In vitro tumor-microenvironment models suggest that EV cargo can influence inflammatory pathways such as NF-κB signaling, promote M2-like macrophage polarization, and modulate T-cell activation. These changes may support a pro-tumor immune milieu characterized by cytokines such as interleukin-6 (IL-6) and C-X-C motif chemokine ligand 8 (CXCL8/IL-8), particularly under stress conditions such as hypoxia [133]. Clinically, inflammatory and immune-suppressive signatures are associated with aggressive TC phenotypes, dedifferentiation, EMT, and reduced radioiodine responsiveness; however, it remains difficult to determine whether EVs are causal mediators or secondary markers of aggressive disease, or both. 

Studies on clinical samples showed that the serum value of EVs miR-655-3p is significantly downregulated in PTC patients [90]. Functionally, preclinical experiments indicate that miR-655-3p can suppress PTC cell viability, chemotaxis, and invasion by targeting C-X-C Motif Chemokine Receptor 4 (CXCR4) and can inhibit M2 macrophage polarization, including reduction in CD163-positive macrophage populations [90]. In contrast, miR-519e-5p, which acts as an oncogenic factor that facilitates distant metastasis, was found to be upregulated in the serum of patients with TC distant metastases [129]. PTC-derived EVs miR-519e-5p promoted a more aggressive tumor phenotype by causing CD8+ T-cell exhaustion and therefore helping in immune escape of the tumor. MiR-519e-5p in CD8+ T cells downregulates Notch Receptor Family 2 (NOTCH2), which inhibits the Notch signaling pathway, leading to CD8+ T-cell exhaustion and allowing the tumor to evade the immune system in distant organs. It was additionally shown that EVs with miR-155 ensure a permissive environment for TC cell growth by modulating the polarization of macrophages toward a tumor-supportive phenotype, typically through the activation of pathways like β-catenin [130]. Therefore, circulating EVs miR-155 is considered a potential non-invasive biomarker for monitoring disease progression and relapse [134]. These findings are coherent with an immune-evasion model, but the clinical observations remain mostly correlative, and the causal immune mechanisms are still based largely on cell-line, immune-cell co-culture, or other preclinical systems.

Therefore, TC-derived EVs offer a biologically plausible framework for immune escape, but the evidence hierarchy remains weighted toward preclinical studies. In vitro findings support EV-mediated modulation of T cells, NK cells, macrophages, and inflammatory pathways; animal models provide limited in vivo plausibility; and clinical studies mainly identify EV cargo associated with aggressive behavior, metastasis, or relapse. But, for clinical application, the strongest near-term use is likely biomarker development rather than therapeutic targeting. 

### 4.6. TC-Derived EVs and Apoptosis

TC EVs exert a dualistic, compartment-specific influence on apoptosis, serving as master regulators of both tumor survival and immune evasion [125]. Within the tumor microenvironment, TC EVs may act as potent anti-apoptotic vectors that safeguard TC cells from programmed cell death and therapy-induced stress. Conversely, when interacting with the host’s immune system, these same vesicles function as pro-apoptotic projectiles, selectively destroying tumor-infiltrating immune cells to enforce immune tolerance. 

In vitro studies on human TC cell lines, such as TPC-1, BCPAP (for PTC), and SW579, 8505C, or ARO (for ATC), have shown that aggressive TC variants pack high concentrations of oncogenic ncRNAs into EVs (e.g., miR-21, miR-146b, miR-222, lncRNA MALAT1), which have then been transferred to the recipient cells [107,125]. Once internalized, this cargo epigenetically represses critical pro-apoptotic tumor suppressors, most notably PTEN (Phosphatase and Tensin Homolog) and Bax (Bcl-2-associated X protein), triggering hyperactivation of the PI3K/Akt/mTOR survival pathway, which upregulates downstream anti-apoptotic executioners like Bcl-2 and enables survival of TC cells. Furthermore, additional stress stimuli (such as hypoxia or chemotherapeutic agents like cisplatin) drastically escalate EV secretion in these cell lines, increasing resistance and rescuing them from apoptosis. While protecting the tumor, TC EVs behave destructively toward infiltrating immune cells. The targeted pro-apoptotic effect helps the tumor evade the host immune response. This systemic immune ablation has been validated using in vitro studies and in vivo animal models [135,136]. It has been shown that TC EVs exploit the extrinsic apoptotic pathway by presenting active death ligands directly on their lipid bilayers, including Fas ligand, TRAI (Tumor necrosis factor-related apoptosis-inducing) ligand, and immune checkpoint proteins like PD-L1 [137]. When these vesicles bind to their respective receptors on cytotoxic CD8+ T-lymphocytes and NK cells, they trigger receptor clustering and the recruitment of FADD (Fas-associated death domain protein) [135]. This activates caspase-8 and caspase-3, systematically forcing these vital anti-tumor effector cells into apoptosis. Furthermore, EV cargo downregulates activating receptors (such as NKG2D) on NK cells, inducing functional exhaustion before cell death. In xenograft mouse models (e.g., injecting human ATC or PTC cell lines into nude or SCID mice), researchers tracked the biodistribution of fluorescently labeled TC EVs. These studies confirmed that circulating EVs accumulate in peripheral immune organs like the spleen and draining lymph nodes, where they drive profound T-cell depletion via apoptosis, mirroring the systemic immune suppression observed in advanced cancer stages.

The translation of these apoptotic mechanisms from bench to bedside remains a major bottleneck in thyroid oncology, characterized by a sharp divide between preclinical promise and actual clinical implementation. Preclinical strategies are strictly focused on breaking this apoptotic asymmetry. Researchers utilize pharmacological inhibitors of EV biogenesis/secretion, such as GW4869, to halt the spread of anti-apoptotic signals within the TME. Additionally, natural compounds like Evodiamine (EVO) have been tested in vitro and in vivo ATC models [136,138]. EVO successfully suppresses the oncogenic EV cargo, driving the cancer cells back into caspase-dependent apoptosis while safeguarding surrounding tissue. Alternate approaches explore the use of engineered mesenchymal stem cell-derived EVs (MSC-EVs) to deliver pro-apoptotic small interfering RNAs directly to refractory PTC cells.

However, significant methodological flaws narrow the understanding of EVs’ influence on apoptosis in TC patients. First, most data rely on immortalized cell lines (in vitro) that have adapted to artificial plastic environments over decades, failing to replicate the architectural complexity and physical stressors of a human thyroid nodule. Second, the current in vivo xenograft models lack a fully intact, syngeneic immune system, which heavily skews observations regarding how TC EVs induce immune-cell apoptosis. Finally, standard isolation protocols frequently co-isolate non-vesicular protein aggregates and non-tumor host EVs, making it incredibly difficult to definitively attribute specific pro- or anti-apoptotic clinical outcomes purely to TC EVs in human patient samples.

Thus, there are no active or completed Phase I–IV clinical trials evaluating therapies that directly target or manipulate TC EV-mediated apoptosis. The clinical utility of these mechanisms is currently confined to liquid biopsy and diagnostics. Clinical observational studies monitoring the plasma of TC patients show that the abundance of circulating, thyroid-specific (TSHR-positive or Tg-positive) EVs [85], or EVs carrying high PD-L1 or anti-apoptotic miRNA signatures, correlates tightly with LNM, radioiodine resistance, and poor disease-free survival. This makes them excellent non-invasive biomarkers for monitoring real-time treatment failure, even if they cannot yet be therapeutically drugged.

### 4.7. TC-Derived EVs in Metastasis and Invasion

EVs facilitate the transition from localized growth to invasive and metastatic stages by carrying the biomolecules that prepare the pre-metastatic niche and stimulate angiogenesis to support sustained tumor expansion. Available evidence supports a role for EVs in TC progression, but the strength of this evidence varies substantially across experimental systems. Many mechanistic conclusions are derived predominantly from in vitro assays and animal models, whereas clinical studies have mostly provided associative biomarker data. Overall, the most consistent data indicate that EVs participate in intercellular communication within the TME, promote angiogenesis, remodel ECM, and may contribute to the formation of a pre-metastatic niche [139,140]. Furthermore, TC EV cargo may promote metastasis and help TC develop resistance to therapy or to become drug-resistant [141,142]. 

Clinical evidence comes from the study showing miR-146b-5p and miR-222-3p to be highly abundant in the serum of PTC patients with LNM and directly correlate with TCs increased invasive potential [94]. Mechanistic support comes mainly from in vitro work showing that miR-146b-5p acts as a suppressor of tumor-suppression genes (SMAD4 and CCDC6), which stimulates tumor cell survival and proliferation, while miR-222-3p may activate the AKT signaling pathway via Phosphatase and Tensin Homolog (PTEN), enabling tumor growth and metastasis. Similarly, EV-associated miR-21-5p has been implicated in angiogenesis and endothelial barrier disruption, particularly under hypoxic conditions, by repressing anti-angiogenic or matrix-associated factors such as TGFBI and COL4A1 [107,109,143]. This leads to “leaky” vessels, which facilitate tumor cell metastasis and provide the nutrients necessary for rapid tumor growth [143]. These findings provide biological plausibility for EV-mediated vascular permeability and metastatic dissemination, but they are largely based on cell culture systems and therefore require confirmation in clinically relevant animal models and prospective patient cohorts.

Evidence from animal models supports the concept that tumor-derived EVs can condition distant tissues, stimulate angiogenesis, and facilitate metastatic colonization; however, these models often rely on selected cell lines, high EV doses, or simplified metastatic settings that may not fully reproduce human TC biology. EV proteins and non-coding RNAs such as periostin, BID, and circRNAs have been proposed as contributors to ECM remodeling, EMT, resistance to cellular stress, and invasive behavior [144,145,146]. Periostin, for example, is biologically plausible as a mediator of matrix remodeling and motility, BID, a pro-apoptotic member of the Bcl-2 protein family, in tumor-derived EVs acts as a critical mediator of intercellular communication, enhancing survival under environmental stress (hypoxia, acidity) and facilitating metastatic progression, while circRNAs may regulate oncogenic signaling by acting as miRNA sponges or act as oncogene in TC progression by promoting cell proliferation, migration, and invasion while inhibiting apoptosis. Nevertheless, the clinical application of these markers remains preliminary: most studies are exploratory, analytical platforms are not harmonized, and thresholds for risk stratification have not been validated across independent populations. 

Therapeutic use of EVs is even less mature. Novel preclinical findings suggest that EVs derived from human dental pulp stem cells (hDPSC-EVs) can fine-tune TC gene networks, specifically targeting Fibronectin 1 (FN1), to selectively inhibit migration and invasion without promoting oncogenic proliferation [141]. Rather than randomly blocking processes, they modify gene networks to revert a pro-metastatic phenotype into a more stable, less mobile state. By suppressing the FN1 gene, these vesicles effectively “disarm” tumor cells, stripping them of their ability to bind to the substrate and migrate toward lymph nodes. Although this provides a rationale for EV-based anti-metastatic strategies, the current evidence is mainly experimental. Understanding these mechanisms is essential for developing novel interventions to intercept tumor dissemination. Potential advantages include biological targeting, low immunogenicity, and the possibility of delivering regulatory RNAs or proteins to tumor cells. Conversely, major concerns include unclear biodistribution, variable cargo composition, manufacturing and storage challenges, risk of unintended pro-tumor signaling, lack of dose standardization, and absence of robust safety and efficacy data in TC patients.

### 4.8. Key EV-Driven Pathways in TC Pathogenesis

Tumor-derived EVs are increasingly implicated in TC progression, but the strength of evidence varies substantially across experimental contexts. Overall, the current literature supports a biologically plausible role for EVs in intercellular communication within the tumor microenvironment; however, most mechanistic conclusions remain derived from in vitro systems and animal models rather than from prospective clinical investigations. Thus, EV-mediated transfer of oncogenic cargo should be interpreted as a promising but still incompletely validated mechanism in human TC.

The available evidence converges on several interacting pathways: EMT and invasion through Wnt/β-catenin, TGF-β/SMAD, SOX4, PAX6, SNAIL/SLUG, ZEB1/2, and loss of E-cadherin; PI3K/AKT/mTOR survival signaling, frequently linked to PTEN suppression and apoptosis resistance; hypoxia and angiogenesis through miR-181a–MLL3/DACT2–YAP–VEGF, miR-21-5p/TGFBI-COL4A1, and miR-210-3p or miR-221-3p networks; metabolic reprogramming via PKM2, PDK4, AKT, and Warburg-like glycolysis; ECM remodeling through CD147, MMP2, MMP9, periostin, and related matrix regulators; and immune escape through PD-L1-positive EVs, HMGB1–mTORC2/AKT/c-MYC signaling, CD8+ T-cell exhaustion, NK-cell suppression, macrophage polarization, and inflammatory NF-κB/IL-6/IL-8 networks. Collectively, these pathways suggest that TC EVs may coordinate several hallmarks of progression: they promote phenotypic plasticity, prepare permissive stromal and vascular niches, suppress anti-tumor immunity, protect malignant cells from apoptosis, and potentially condition distant sites for metastasis. However, the current evidence should be interpreted as biologically plausible rather than clinically definitive. In particular, EV-specific causality is difficult to prove when the same molecules are also present as free-circulating nucleic acids, protein complexes, or tumor tissue markers.

Nevertheless, several limitations temper the clinical interpretation of this field. First, EV isolation, characterization, and normalization methods differ markedly between studies, making cross-study comparison difficult. Second, many reports use small patient cohorts or lack longitudinal sampling, external validation, and adjustment for established clinicopathological risk factors. Third, mechanistic studies often infer EV-specific effects from purified vesicle preparations, although EV preparations may contain co-isolated proteins, lipoproteins, or RNA–protein complexes that may contribute to observed biological activity. Fourth, mechanistic conclusions frequently rely on in vitro assays that cannot fully reproduce the specific organization, antigen specificity, stromal complexity, vascularization, immune heterogeneity and endocrine context of human TC. Five, many mechanistic experiments use overexpression or knockdown systems, and limited TC cell lines, which may exaggerate pathway dependence. Six, many studies assume process of interest from changes in selected markers rather than from comprehensive functional assays. Seven, most functional experiments rely on supraphysiological EV concentrations or manipulated cargo expressions, which may not reflect endogenous EV signaling in TC patients. Eight, in vivo animal models provide useful biological support but only partial approximation of human TC pathogenesis, as they cannot recapitulate human thyroid-specific immunity or the effects of prior surgery, radioiodine, kinase inhibitors, or immune-checkpoint therapy. Nine, TC is biologically heterogeneous; findings from PTC, FTC, DTC, or ATC models should not be generalized across all histological subtypes or stages, as TC subtypes differ markedly in immune contexture: PTC is often relatively immune-cold, whereas PDTC and ATC may show stronger inflammatory and immunosuppressive features. Consequently, EV biomarkers must be validated separately by subtype, disease stage, and treatment setting.

From a clinical perspective, the main argument for EV application is their potential as minimally invasive biomarkers. TC-derived EVs may reflect tumor-specific molecular information and enable monitoring of dynamic processes such as hypoxia, glycolytic adaptation, aggressive phenotypic shifts, and response or resistance to immunotherapy, particularly in advanced PDTC or ATC. EV cargo profiling may complement tissue immunohistochemistry, cytology, genomic testing, cytokine profiling, and imaging by providing real-time information from blood or other accessible biofluids. EVs could also support postoperative surveillance, especially in patients with indeterminate nodules, recurrent disease, radioiodine-refractory tumors, or ATC. Over time, TC EV cargo may help identify therapeutic vulnerabilities or provide a platform for targeted delivery. However, important limitations remain: EV isolation, normalization and cargo quantification protocols are not standardized; clinically validated cut-offs are lacking; most studies are small, retrospective, subtype-limited, or experimentally focused; there is unclear biodistribution and safety for therapeutic EVs; and the possibility that blocking EV release could affect normal endocrine, immune, and stromal communication. 

## 5. EVs as Biomarkers in Thyroid Cancer

The unique phospholipid bilayer of EVs protects their molecular cargo from enzymatic degradation in circulation, thereby preserving a stable “fingerprint” of the physiological state of thyroid tumors. EV cargo is involved in regulating diverse pathophysiological processes in TC and may therefore provide useful targets for adjunctive diagnosis, prognostic assessment, and targeted therapy. Accordingly, growing evidence supports the diagnostic, prognostic, and therapeutic biomarker potential of EVs in patients with TC.

**Figure 3:**
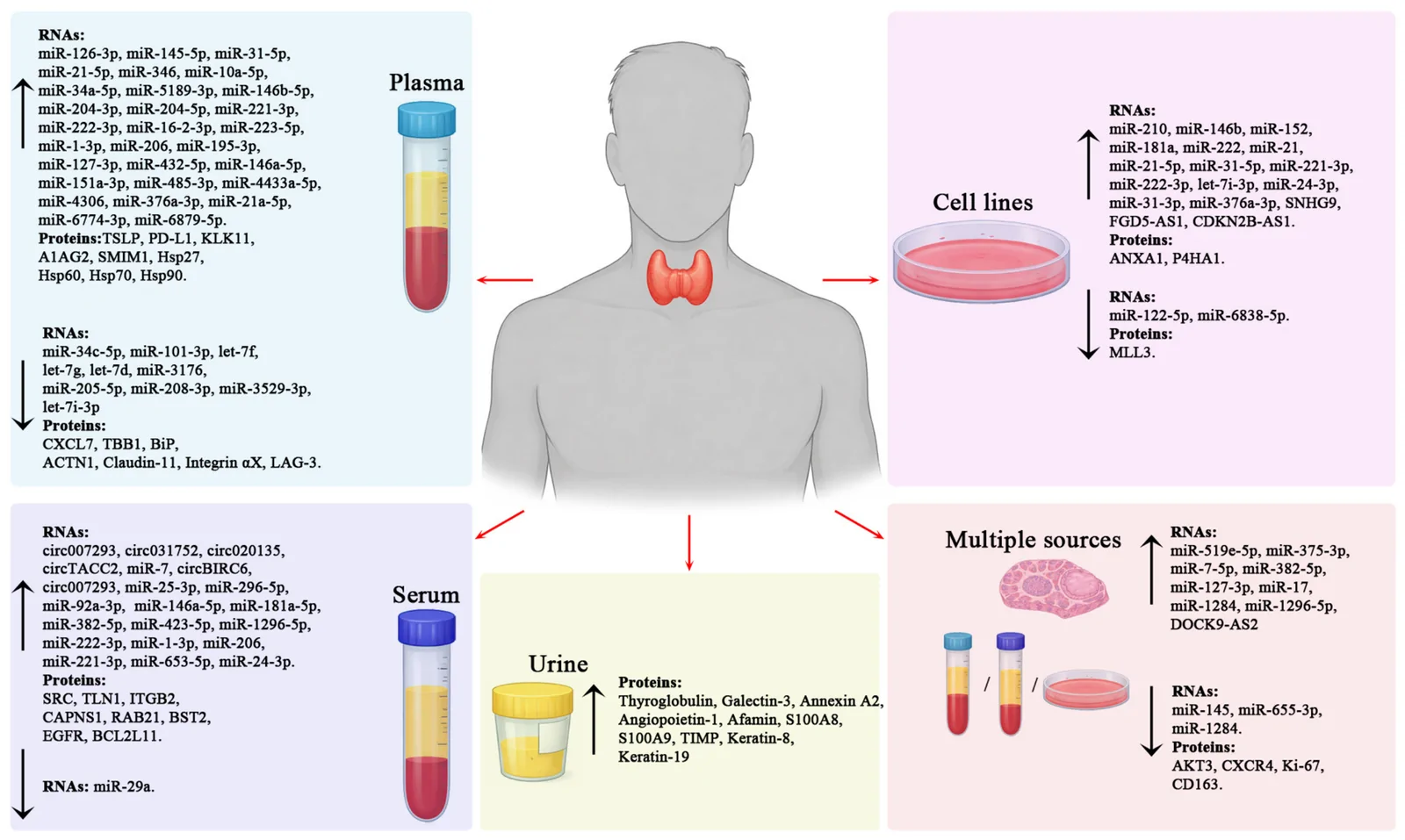
Potential molecular biomarkers identified in thyroid cancer-derived extracellular vesicles (EVs) from different biological sources. Biomarkers reported to be upregulated and downregulated are indicated by ↑ and ↓, respectively. ACTN1, actinin alpha 1; Afamin, afamin; AKT3, AKT serine/threonine kinase 3; ANXA1, annexin A1; ANXA2, annexin A2; ANGPT1, angiopoietin-1; BCL2L11, BCL2-like protein 11 (BIM); BiP, binding immunoglobulin protein; BST2, bone marrow stromal antigen 2; CAPNS1, calpain small subunit 1; CD, cluster of differentiation; circRNA, circular RNA; CXCL7, C-X-C motif chemokine ligand 7; CXCR4, C-X-C chemokine receptor 4; DOCK9-AS2, DOCK9 antisense RNA 2; EGFR, epidermal growth factor receptor; EVs, extracellular vesicles; FGD5-AS1, FYVE, RhoGEF and PH domain containing 5 antisense RNA 1; Hsp, heat shock protein; ITGB2, integrin beta 2; Ki-67, marker of cellular proliferation; KLK11, kallikrein-related peptidase 11; LAG-3, lymphocyte activation gene 3; lncRNA, long non-coding RNA; miRNA (miR), microRNA; MLL3, myeloid/lymphoid or mixed-lineage leukemia protein 3 (KMT2C); P4HA1, prolyl 4-hydroxylase subunit alpha 1; PD-L1, programmed death-ligand 1; RAB21, Ras-related protein Rab-21; S100A8, S100 calcium-binding protein A8; S100A9, S100 calcium-binding protein A9; SMIM1, small integral membrane protein 1; SNHG9, small nucleolar RNA host gene 9; SRC, SRC proto-oncogene, non-receptor tyrosine kinase; TBB1, tubulin beta-1 chain; TIMP, tissue inhibitor of metalloproteinases; TLN1, talin-1; TSLP, thymic stromal lymphopoietin.

Early studies evaluating EVs as biomarkers were primarily based on cell culture experiments. Until recently, however, EVs have attracted increasing interest as stable components of liquid biopsy, offering a non-invasive alternative to traditional fine-needle aspiration (FNA). Consequently, most recent studies have focused on EVs isolated from patient plasma or serum, while urinary EVs have also been explored as an additional non-invasive source (overviewed in Figure 3 and Supplementary Table S1).

### 5.1. Plasma-Derived EV Cargo as Biomarkers

Liquid biopsy has emerged as a promising non-invasive approach for cancer diagnosis, with EVs representing one of its most biologically stable and informative components. Plasma is commonly defined as the liquid component of blood that carries proteins and other circulating molecules. Similarly, the cell-free plasma-like fraction or supernatant obtained from an FNA sample may contain tumor-derived EVs, soluble proteins, metabolites, free nucleic acids, cytokines, and other locally released molecules from thyroid tissue and the surrounding tumor microenvironment, making it a clinically accessible source of tumor-related molecular information. In TC, plasma-derived EVs have been the most extensively investigated as carriers of diagnostic biomarkers, particularly miRNAs, which represent the most studied and clinically promising EV cargo (Supplementary Table S1).

#### 5.1.1. Plasma-Derived EV Cargo in TC Diagnosis

A major clinical challenge in thyroid oncology is the differentiation between benign and malignant thyroid nodules. In this context, multiple studies have confirmed that plasma-derived EV miRNAs can be used as a successful diagnostic tool [147,148]. Plasma-derived EV miRNAs show strong potential as non-invasive biomarkers for distinguishing PTC from nodular goiter (NG), especially in indeterminate cases. Among candidates identified using NGS, miR-5189-3p showed the best diagnostic performance (Area Under the Curve, AUC 0.951). The authors highlight that a combined EVs miRNA panel may significantly improve diagnostic accuracy compared to single markers or conventional methods, although further validation is still required [147]. Regarding indeterminate thyroid nodules, Ahmed et al. demonstrated that circulating large EVs may serve as potential diagnostic biomarkers [149]. Using a multi-platform omics approach, the authors further identified distinct EV-associated molecular signatures capable of differentiating benign from malignant lesions. Among patients with Bethesda IV thyroid nodules, circulating EV-derived miRNA profiles discriminated malignant from benign lesions, with miR-195-3p being upregulated and several miRNAs, including miR-205-5p and let-7i-3p, being downregulated in malignant nodules, highlighting their diagnostic potential. Using RT-qPCR, Delcorte et al. showed that miR-146b-5p and miR-21a-5p were significantly enriched in plasma-EVs from PTC patients, while no significant differences were detected in bulk plasma, highlighting EV-associated miRNAs as more promising diagnostic biomarkers and emphasizing that validation in larger cohorts is required [148]. Similarly, Wang et al. identified a three-miRNA plasma signature (miR-346, miR-10a-5p, and miR-34a-5p) capable of effectively distinguishing PTC from NG and healthy controls [150]. Notably, the combined expression profile showed higher diagnostic accuracy than individual miRNAs, further supporting the value of multi-miRNA panels as non-invasive biomarkers. Also, a study by Liang demonstrated that a panel of plasma-derived EV miRNAs can effectively differentiate benign from malignant thyroid nodules [151]. Specifically, miR-16-2-3p and miR-223-5p were upregulated, and miR-34c-5p and miR-101-3p were downregulated in PTC compared to benign thyroid nodules, distinguishing nodules from healthy controls. Furthermore, D’Amico et al. provided proof-of-concept evidence that plasma-derived EV miR-1-3p, miR-206, and miR-221-3p are upregulated in PTC patients, compared to controls [152]. In distinguishing between benign and malignant thyroid nodules, Zabegina et al. demonstrated that let-7 miRNA derived from TPO^+^ EV may serve as a more thyroid-specific biomarker [95]. Dai et al. identified 41 candidate EV miRNAs by small RNA sequencing, of which miR-376a-3p, miR-4306, miR-4433a-5p, and miR-485-3p were significantly upregulated in PTC compared to both healthy controls and benign nodules [153]. Among them, miR-485-3p and miR-4433a-5p showed the best diagnostic performance, with the highest AUC values. A study by Samsonov and associates showed that plasma EVs miR-31 and miR-21 have diagnostic potential for distinguishing PTC and FTC from benign tumors, while comparative assessment of miR-21 and miR-181a-5p enabled discrimination between PTC and FTC with high sensitivity and specificity [93]. Circulating lEVs showed promising diagnostic potential for indeterminate thyroid nodules, with distinct molecular signatures differentiating benign from malignant lesions, although further validation is needed [149]. Circulating sEV-associated miRNAs demonstrated the strongest translational potential among the investigated circulating RNA species for differentiating FTC from FTA. More precisely, the authors developed a five-miRNA classifier including miR-127-3p, miR-223-5p, miR-432-5p, miR-146a-5p, and miR-151a-3p, which achieved high diagnostic accuracy in both the training cohort (AUC 0.924) and the independent multicentre validation cohort (AUC 0.844) [154]. Importantly, the classifier improved the preoperative identification of FTC and could complement ultrasound and cytology in patients with indeterminate follicular thyroid nodules. 

In a study by Yu et al. focused on the diagnostic and monitoring potential of single-vesicle analysis, EpCAM-positive (EPCAM^+^) plasma-derived small EVs were identified as sensitive and specific biomarkers for TC [155]. Their levels significantly decreased after surgery, further supporting the utility of high-resolution single-vesicle analysis for both early diagnosis and postoperative monitoring. Bavisotto et al. demonstrated that molecular chaperones (Hsp27, Hsp60, Hsp70, and Hsp90) are differentially expressed across normal, benign, and malignant thyroid tissues and are also detectable in circulating EVs [72]. Large EV-derived protein profiling revealed differential expression of several proteins, including upregulation of Kallikrein-Related Peptidase11 (KLK11), Alpha-1-acid Glycoprotein 2 (A1AG2), and Small Integral Membrane Protein 1 (SMIM1) in malignant nodules, highlighting the diagnostic utility of EV-associated proteins in patients with indeterminate thyroid cytology [149].

#### 5.1.2. Plasma-Derived EV Cargo in TC Prognosis

Following their emerging role in the diagnosis of PTC, EVs and their molecular cargo have also attracted considerable attention as potential prognostic biomarkers. As they can help in disease detection, circulating EVs may provide valuable information on tumor behavior, progression, and metastatic potential. Their association with clinicopathological features and disease outcomes highlights their potential utility in risk stratification and in guiding clinical management of patients with PTC. For instance, miR-485-3p was associated with aggressive clinicopathological characteristics, suggesting a role in both the diagnosis and prognostic stratification of PTC patients [153]. The study conducted by Jiang demonstrated that plasma-derived EVs miR-146b-5p and miR-222-3p were significantly associated with LNM in patients with PTC. Higher circulating levels of these miRNAs were detected in patients with metastatic disease compared with those without LNM [94]. These results indicate that EVs miR-146b-5p and miR-222-3p may represent promising non-invasive biomarkers for predicting LNM, potentially improving risk stratification and clinical decision-making for patients with PTC. The study by Chen identified plasma-derived EVs miR-6774-3p and miR-6879-5p as significantly elevated in PTC patients with LNM [156]. Their combined diagnostic performance showed high accuracy for distinguishing metastatic from non-metastatic disease (AUC 0.914), indicating their potential for early detection of LNM in PTC. More recently, a study investigating plasma-derived thyroid EVs identified TSHR as a promising marker for the enrichment of thyroid-specific EVs and suggested that EV-associated Tg may complement conventional monitoring approaches in recurrent TC, particularly in patients with anti-Tg antibodies, where vesicular encapsulation may protect Tg from antibody interference [85]. Furthermore, plasma-derived EV PD-L1 was associated with tumor T stage and immunosuppressive activity in pediatric PTC. These findings suggest that EV PD-L1 may serve as a prognostic biomarker of tumor progression and contribute to immune evasion through suppression of CD8+ T-cell activation [105].

#### 5.1.3. Plasma-Derived EV Cargo in TC Therapy

Beyond their diagnostic and prognostic value, EV-associated molecules may potentially appraise treatment response and resistance, supporting a more personalized therapeutic approach in TC. However, available evidence remains limited as only a few studies have examined plasma-derived EV cargo in relation to TC therapy (Supplementary Table S1). Li et al. reported that plasma-derived EV miR-1296-5p was associated with radioiodine-refractory PTC, suggesting its potential as a non-invasive biomarker for treatment resistance and therapeutic stratification [157]. This observation was further supported by mechanistic findings in radioiodine-refractory TC cell lines and tumor tissue specimens, where miR-1296-5p was linked to Sodium/Iodide Symporter (NIS)-related pathways [157]. Another study detected increased levels of EV-associated lncRNA DOCK9-AS2 in plasma from TC patients [99]. In vitro functional experiments suggested that cancer stem cell-like cell-derived EV lncRNA DOCK9-AS2 may promote PTC progression through activation of the Wnt/β-catenin pathway. Although these findings indicate possible therapeutic relevance, they remain preliminary and require independent longitudinal validation before clinical implementation.

The limited direct evidence should not be interpreted as an absence of biological relevance, but rather as a translational gap between expanding EV research and clinically validated EV-based therapeutic applications in TC. Several factors may explain this gap. TC is biologically heterogeneous, making it difficult to design unified EV-based therapeutic studies across different histological and molecular subtypes. In addition, plasma is analytically complex because circulating EVs originate from platelets, leukocytes, endothelial cells, immune cells, and other tissues; consequently, tumor-derived EVs may represent only a minor fraction of the total EV pool. This complicates the identification of thyroid tumor-specific cargo. Furthermore, EV isolation, purification, and characterization methods remain insufficiently standardized, limiting reproducibility and comparability between studies. Finally, the possibility that some relevant studies were accidentally missed during the literature search cannot be excluded. Overall, the scarcity of direct evidence should be framed as a clinically meaningful knowledge gap, indicating that plasma-derived EV cargo in TC has not yet progressed from biomarker-oriented research to validated therapy-guiding or therapy-delivering application.

### 5.2. Serum-Derived EV Cargo as Biomarkers

Both serum- and plasma-derived EVs have gained attention as potential sources of non-invasive biomarkers in TC, but they are not interchangeable. Plasma is obtained from anticoagulated blood and more closely reflects circulating EVs present in vivo, whereas serum is obtained after coagulation, during which platelet activation can release additional platelet-derived EVs and alter the overall EV profile. Thus, serum-derived EV cargo may still provide clinically useful biomarker information, but its interpretation requires caution because pre-analytical clotting-related changes may influence EV quantity and molecular composition. Overall, the clinical value of serum-derived EV cargo in TC remains investigational, but it requires careful methodological standardization and validation before routine diagnostic or prognostic use.

#### 5.2.1. Serum-Derived EV Cargo in TC Diagnosis

A pilot study by Yang and collaborators, conducted on a limited cohort of three PTC and three benign thyroid goiter serum samples, identified altered EV circRNA profiles associated with PTC [103]. Using EV isolation, transmission electron microscopy, high-throughput sequencing, and qRT-PCR validation, 22 differentially expressed circRNAs were detected, with circ007293, circ031752, and circ020135 confirmed as the main dysregulated candidates, supporting the potential of serum EV circRNAs as diagnostic biomarkers and warranting further investigation in larger cohorts. Similarly, Zou et al. identified a three- miRNA signature consisting of miR-25-3p, miR-296-5p, and miR-92a-3p [158]. Following qPCR-based screening, training, testing, and external validation, this panel demonstrated strong diagnostic performance and accurately discriminated PTC from benign goiter, highlighting its promise as a non-invasive diagnostic tool. Additional evidence for the diagnostic utility of serum EV-derived miRNAs was provided by Capriglione et al., who identified four differentially expressed miRNAs, namely miR-24-3p, miR-146a-5p, miR-181a-5p, and miR-382-5p [159]. Although these miRNAs successfully distinguished PTC patients from healthy controls, no consistent association with LNM was observed, suggesting that their value lies primarily in diagnosis rather than disease stratification. Advances in biosensing technologies have further expanded the diagnostic potential of EV-associated miRNA. In this context, Gao et al. analyzed EVs miR-222-3p using a highly sensitive electrochemiluminescence biosensor and demonstrated its association with the B-Raf Proto-Oncogene, Valine (V) to Glutamic Acid (E) mutation at codon 600 (BRAFV600E), supporting its utility in improving TC diagnosis and assessment of disease progression [160]. Protein-based EV biomarkers have also emerged as promising diagnostic candidates. For example, Kawakami et al. identified RAB21 as a highly enriched protein in serum-derived EVs from FTC patients, distinguishing FTC from FTA and suggesting a role in tumor progression through the regulation of cell migration [121].

#### 5.2.2. Serum-Derived EV Cargo in TC Prognosis

Besides their diagnostic utility, several serum EV-derived molecules have also been linked to disease aggressiveness, recurrence, and metastatic potential, highlighting their value as prognostic biomarkers in TC.

In this regard, Wen et al. demonstrated that serum EVs miR-29a possesses both diagnostic and prognostic value in PTC [161]. Its expression was significantly reduced in PTC patients compared with controls, while lower levels were associated with recurrence, adverse clinicopathological characteristics, and shorter overall survival [161]. Mechanistically, EVs circ007293 was shown to drive EMT and enhance the invasive, migratory, and proliferative capacity of PTC cells through the miR-653-5p/PAX6 pathway, supporting its utility as a biomarker of disease progression [101]. Likewise, Peng et al. identified a distinct EVs circRNA signature associated with LNM in PTC [102]. Through NGS-based serum profiling and bioinformatics analyses, several circRNA–miRNA–mRNA regulatory axes, including circTACC2–miR-7– Epidermal Growth Factor Receptor (EGFR) and circBIRC6–miR-24-3p–B-Cell Lymphoma 2-Like Protein (11BCL2L11), were implicated in metastatic progression, suggesting their value as biomarkers of metastatic disease. A broader EV-associated marker, Tumor-Associated Calcium Signal Transducer 2 (TACSTD2), was found to be elevated in several malignancies, including TC, indicating its potential as a biomarker of tumor progression [162]. Among protein-based prognostic biomarkers, Bone Marrow Stromal Cell Antigen 2 (BST2) emerged as a particularly promising candidate. Elevated BST2 levels in serum-derived sEVs were associated with LNM in papillary thyroid microcarcinoma (PTMC), while functional studies demonstrated that sEV-derived BST2 promotes tumor cell proliferation, migration, lymphangiogenesis, and metastatic progression [163]. Proteomic analyses performed by Luo et al. further revealed that serum EVs from PTC patients with LNM exhibit a distinct signature enriched in integrin-associated and metastasis-related proteins [70]. These EVs also enhanced TC cell invasiveness, emphasizing their association with metastatic behavior. Additional support for the clinical relevance of serum EV-derived miRNAs was provided by D’Amico, who demonstrated elevated levels of miR-1-3p, miR-206, and miR-221-3p in PTC patients compared with benign goiter controls [152]. Their postoperative decrease reinforced their value for disease monitoring.

#### 5.2.3. Serum-Derived EV Cargo in TC Therapy

Some EV-derived biomarkers appear to play an active role in tumor progression rather than merely reflecting disease status, making them attractive candidates for therapeutic targeting. However, current evidence remains preliminary and requires stronger mechanistic and clinical validation.

In line with this concept, Ye et al. identified serum EVs miR-423-5p as a molecule with both biomarker and therapeutic potential in PTC [119]. Elevated circulating levels were observed in the patients, while functional assays demonstrated that EV-mediated transfer of miR-423-5p promoted TC cell migration and invasion. Conversely, silencing of miR-423-5p suppressed these effects, indicating therapeutic potential in TC. Similar evidence has been reported for EV miR-17-5p, which was elevated in the serum of TC patients with lung metastases [88]. Mechanistic studies demonstrated that miR-17-5p remodeled the pulmonary microenvironment by activating pro-inflammatory signaling in lung fibroblasts, thereby promoting tumor growth and metastatic colonization [88]. In addition, miR-222-3p was found to be significantly enriched in serum EVs from TC patients, suggesting possible therapeutic relevance [160]. 

Despite these promising findings, the field remains limited by small cohorts, predominantly associative designs, heterogeneous EV isolation and detection methods, as well as limited in vivo or longitudinal validation. Most candidate EV cargo molecules have not yet been shown to drive TC progression causally when targeted therapeutically. Before clinical translation, standardized protocols, mechanistic studies, independent validation cohorts, and well-designed longitudinal or interventional trials are required to establish biological relevance, safety, efficacy, and clinical utility.

### 5.3. Urinary-Derived EV Cargo as Biomarkers

In addition to serum- and plasma-derived EVs, urinary EVs have emerged as a promising source of non-invasive biomarkers given the simplicity of sample collection. Recent studies have explored the prognostic potential of urinary EV proteins in DTC. In this context, a study by Wang investigated the potential prognostic value of urinary EV proteins in DTC [164]. Elevated levels of TIMP and angiopoietin-1 were associated with LNM, suggesting their potential utility in preoperative risk stratification and identification of high-risk TC patients. 

In addition to their potential role in preoperative risk stratification, urinary EV proteins have also been investigated as biomarkers for postoperative monitoring and recurrence surveillance in TC. A longitudinal study by Wang et al. evaluated a panel of urinary EVs proteins and peptides after thyroidectomy, including Tg, galectin-3, angiopoietin-1, TIMP, and keratin-19, and found that stable postoperative levels were associated with a lower risk of TC recurrence during long-term follow-up [106]. As no disease recurrence was observed during the study period, the authors suggested that biomarker concentrations remaining within these ranges may be indicative of a favorable clinical course and a lower likelihood of recurrence. Expanding on these findings, another study evaluated urinary EVs Tg as a marker of recurrent disease and reported its increasing levels even in patients with undetectable serum Tg after surgery and radioactive iodine ablation [74]. These results suggest that urinary EV biomarkers may not only contribute to recurrence risk assessment but could also improve the early detection of recurrent or residual disease, potentially complementing or even surpassing conventional serological monitoring strategies.

### 5.4. Cell Line-Derived EV Cargo and Biomarker Discovery

While studies investigating EV-associated biomarkers in biofluids such as plasma, serum, and urine have demonstrated their potential clinical utility, research based on TC cell lines has played a crucial role in elucidating the molecular composition and biological functions of EV cargo. Cell line-derived EVs provide a controlled experimental model that enables the identification of tumor-specific RNAs, miRNAs, proteins, and other biomolecules released by malignant thyroid cells, thereby facilitating the discovery of candidate biomarkers and therapeutic targets. Importantly, cell line-based studies allow the identification of tumor-specific EV cargo without the confounding effects of non-tumor cells and circulating vesicles present in patient-derived biofluids.

#### 5.4.1. Cell Line-Derived EV Cargo in TC Diagnosis and Prognosis

One of the earliest studies investigating EV-associated biomarkers in TC demonstrated that EVs released by TPC-1 cells (PTC cell line wild-type for BRAFV600E) were enriched in miR-146b and miR-222, two miRNAs previously associated with tumor aggressiveness and recurrence [86]. These findings provided initial evidence that TC-derived EVs may serve as carriers of clinically relevant molecular signatures and represent a potential source of biomarkers, in this case for monitoring disease recurrence. Further evidence supporting the biomarker potential of EV miRNAs was provided by comparative profiling studies of PTC cell lines [82]. Five EV miRNAs (miR-21-5p, miR-31-5p, miR-221-3p, miR-222-3p, and let-7i-3p) were found to be significantly deregulated in the EVs of tumor cells compared with non-tumorigenic thyroid cells. These findings provided proof-of-concept that EV miRNAs may serve as a valuable source of novel diagnostic biomarkers for TC [83]. Notably, miR-31-5p, miR-222-3p, and let-7i-3p were associated with the more aggressive K1 cell line (PTC cell line mutant for BRAFV600E), suggesting their potential utility as both diagnostic and prognostic biomarker candidates. Importantly, elevated levels of EVs miR-21-5p were also detected in the serum of patients with PTC, linking in vitro findings with clinical observations. These results suggest that EVs miR-21-5p may represent a promising diagnostic and prognostic biomarker associated with tumor angiogenesis and microenvironmental remodeling. A similar role of hypoxia-induced EV miRNAs was reported for miR-181a in PTC. Under hypoxic conditions, PTC cells exhibited increased expression of miR-181a accompanied by enhanced EV secretion [87]. Another hypoxia-responsive miRNA identified in TC-derived EVs is miR-210-3p. Increased expression of miR-210-3p was observed in ATC cells under hypoxic conditions, and the miRNA was detected in association with EVs and Argonaute-2-containing complexes [92]. Although clinical validation in patient-derived samples is still lacking, these findings suggest that EVs miR-181a may represent a promising candidate prognostic biomarker and therapeutic target associated with hypoxia-driven tumor aggressiveness. 

Along with miRNAs, EVs have also emerged as important carriers of functional lncRNAs. It was shown that EV-derived FGD5-AS1 promotes endothelial cell proliferation, migration, angiogenesis, and vascular permeability through activation of the miR-6838-5p/VAV2 axis [97]. Although the clinical utility of FGD5-AS1 EVs has not yet been validated, its pro-angiogenic and pro-metastatic effects suggest its potential as a candidate prognostic biomarker and therapeutic target in TC. CDKN2B-AS1 was found to be highly expressed in cancer stem cell-like cell-derived EVs and promoted tumor growth, migration, invasion, EMT, and metastasis [98]. These observations position CDKN2B-AS1 as a key regulator of TC progression with possible implications for prognosis and therapeutic intervention. Another lncRNA identified in EVs of PTC was SNHG9 [96]. Elevated SNHG9 expression was detected in both PTC cells and their EVs and was associated with tumor size. Although primarily mechanistic in nature, these findings suggest that SNHG9 may represent a potential diagnostic and prognostic biomarker candidate warranting further clinical investigation.

In addition to ncRNAs, transcriptomic profiling of EVs released by PTC cells further expanded the spectrum of potential EV-associated biomarkers. Comparative analysis revealed thousands of differentially expressed transcripts between tumor-derived EVs and their parental cells, indicating selective RNA packaging into EVs. Notably, Sialic acid-binding Immunoglobulin-like Lectin 10 (SIGLEC10), SIGLEC11, and Keratin-Associated Protein 5-3 (KRTAP5-3) transcripts were exclusively enhanced in tumor-derived EVs and were associated with cancer-related pathways.

Besides the RNAs, studies using TC cell lines have identified EV-associated proteins as potential biomarker candidates. Using a shotgun nanoLC-MS/MS approach, researchers identified a distinct protein signature in EVs released by ATC cells. The detection of multiple tumor-specific proteins in EVs indicates that EV-associated proteins could serve as a source of novel diagnostic biomarkers for TC [64]. Further proteomic characterization of EVs derived from FTC (FTC-133) and ATC cell lines (8305C) revealed enhancement of proteins involved in cell adhesion, migration, drug resistance, and immune evasion. Integration of proteomic data with The Human Protein Atlas identified several proteins with potential diagnostic and prognostic relevance. Importantly, cancer-derived EV emphasized the migratory capacity of recipient thyroid cells, while EV from 8305C cells additionally increased cell viability, supporting the biological significance of the identified protein cargo. These findings suggest that EV-associated proteins may serve not only as candidate biomarkers but also as mediators of TC progression [165]. Among EV-associated proteins, ANXA1 has emerged as a particularly interesting candidate. ANXA1-positive EVs transferred from TC cells to normal thyroid follicular epithelial cells promoted proliferation, invasion, EMT, and tumor growth in vivo [166]. These observations indicate that EV-associated ANXA1 contributes to TC progression and may represent both a promising prognostic biomarker candidate and a potential therapeutic target. 

The complexity of EV cargo is further influenced by interactions within the tumor microenvironment. Co-culturing of TC cells and fibroblasts led to the creation of EVs with a different protein composition, which mainly participates in the remodeling of the extracellular matrix, shaping the tumor microenvironment [69]. These EVs promoted MMP2 activation and displayed functional properties associated with tumor progression. Notably, MMP2-associated protein networks enabled discrimination between EVs derived from tumoral and non-tumoral microenvironments, representing novel diagnostic biomarker candidates.

#### 5.4.2. Cell Line-Derived EV Cargo in TC Therapy

Together with their diagnostic and prognostic applications, EV-derived molecules have attracted growing interest as mediators of therapeutic response and resistance in TC. Evidence from cell line-based studies indicates that EV cargo can modulate pathways involved in drug sensitivity, tumor adaptation, and disease progression, corroborating its potential applicability as a source of predictive and therapy-monitoring biomarkers. Additionally, the direct functional involvement of several EV-associated molecules in these processes has revealed their contribution as promising targets for therapy. The therapeutic potential of EV-associated lncRNAs has been explored in TC stem-like cell-derived EVs. Among the transferred molecules, linc-ROR was shown to induce EMT in recipient thyroid cells. Importantly, inhibition of linc-ROR diminished invasive properties, providing a rationale for exploring this lncRNA in the development of targeted therapeutic approaches [100]. Experimental findings from TC cell models suggest that EV release is closely linked to oncogenic signaling activity. Mitogen-Activated Protein Kinase (MAPK) pathway inhibition alters EV release, suggesting that EV secretion reflects adaptive responses to targeted therapy [167]. The potential of EV-derived molecules for monitoring therapeutic response was demonstrated in a recent study investigating vandetanib treatment in ATC [168]. It was concluded that, in a preclinical ATC model, vandetanib treatment generated a distinct EV-associated RNA signature, supporting the feasibility of EV-derived transcripts as biomarkers of therapeutic response. In lenvatinib-resistant TC cells, increased glycolytic activity was associated with altered expression of glycolysis-related lncRNAs in both cells and their EVs. In fact, treatment with the Hsp90 inhibitor KU757 reversed these changes, suggesting that EV-associated lncRNAs may contribute to metabolic adaptations underlying lenvatinib resistance and represent potential therapeutic targets [169]. Transcriptomic and proteomic analyses identified an eight-RNA signature in EV-associated cell-free RNA that reliably distinguished vandetanib-treated cells from untreated controls. As these biomarkers could be detected and quantified by RT-qPCR, they represent promising candidates for liquid biopsy-based monitoring of treatment response, although further clinical validation is required.

Parallel to their potential role in monitoring therapeutic response, EV-associated molecules have also emerged as promising therapeutic targets. Several studies have demonstrated that modulation of specific EV-derived miRNAs and lncRNAs can directly influence TC cell proliferation, invasion, migration, and treatment resistance. Thus, EVs miR-152 has been shown by Tang and collaborators to be implicated in the suppression of TC progression [89]. Transfer of miR-152 via bone marrow mesenchymal stem cell-derived EVs inhibited proliferation, migration, and invasion of TC cells by targeting Dipeptidyl Dipeptidase 4 (DPP4), suggesting a potential therapeutic application.

Moreover, their role as carriers of biologically active molecules is not limited to intercellular communication, as their potential exploitation as delivery vehicles for therapeutic agents has also been investigated. EV-mediated delivery of SCD-1 siRNA suppressed proliferation and promoted apoptosis in ATC cells, providing a basis for EV-based therapeutic strategies for aggressive thyroid malignancies [104]. Zhang et al. demonstrated that targeted delivery of CRISPR/Cas9 via Tumor-Homing Peptide 1 (TMTP1)-modified EVs improved vemurafenib sensitivity in BRAFV600E-mutant ATC, supporting the potential of EV-based gene therapy for resistant disease [170]. Also, EVs from alternative biological sources, such as mesenchymal stem cells, have shown therapeutic potential, further expanding the clinical applications of EV-based approaches in TC. Namely, EVs derived from human immature dental pulp cells have been shown to have anti-tumor effects in PTC, supporting their potential use as a novel therapeutic approach [138].

Overall, in vitro studies provide a valuable mechanistic framework, but their clinical usability should be interpreted cautiously. These models have been essential for identifying EV-associated RNAs, proteins, and functional cargo capable of influencing proliferation, invasion, EMT, angiogenesis, metabolic reprogramming, and treatment resistance in TC. They also allow controlled testing of EV-mediated biomarker release, therapeutic response signatures, and experimental delivery platforms. However, this strength is also their main limitation: simplified cell culture systems cannot adequately reproduce the spatial, immunological, stromal, vascular, endocrine, and mechanical complexity of thyroid tumors in vivo. Many reported effects depend on selected cell lines, supraphysiological EV concentrations, non-standardized isolation procedures, and short-term functional assays, which may overestimate biological relevance and therapeutic applicability. Therefore, in vitro EV findings should be considered hypothesis-generating rather than directly actionable. Their true value lies in prioritizing candidate mechanisms and biomarkers for validation in real model systems, including patient-derived models, co-cultures, organoids, animal studies, longitudinal liquid biopsy cohorts, and ultimately interventional clinical studies. Without such validation, EV-based signatures and therapeutic strategies in TC remain promising but insufficiently mature for routine clinical decision-making.

### 5.5. EV Biomarkers Across Multiple Biological Sources

In addition to studies focusing on individual sample types, several investigations have analyzed EV cargo across multiple biological sources. These studies offer a broader perspective on EV-associated molecules and may facilitate the identification of biomarkers with greater reproducibility and clinical relevance in TC. 

A comprehensive analysis of miR-145 across multiple sample types, including TC cell lines, FNA specimens, serum, and serum-derived EVs, highlighted its potential clinical relevance in TC. Boufraqech et al. showed that miR-145 expression was reduced in tumor cells and tissues, and that circulating and EV levels were significantly elevated in patients with TC, suggesting active secretion by malignant cells [91]. Functional studies have shown that miR-145 inhibits tumor cell proliferation, invasion, migration, and angiogenesis through regulation of the Phosphoinositide 3-Kinase (PI3K)/AKT signaling pathway and direct targeting of AKT3. At the same time, the authors showed that miR-145 is secreted through serum EVs and can be detected in circulation, and that, in the analysis of indeterminate FNA samples, it showed a high negative predictive value (NPV 92%) for distinguishing benign from malignant nodules, indicating its potential as an auxiliary diagnostic biomarker. 

A similar pattern was observed for miR-1284, which exhibited reduced expression in TC tissues and cells but elevated levels in the peripheral blood of affected patients. In vitro experiments further demonstrated that miR-1284 suppressed proliferation and migration while promoting apoptosis in TC cells [171]. Moreover, PTC-derived EV miR-519e-5p was shown to promote distant metastasis by enhancing tumor aggressiveness and facilitating immune evasion through modulation of CD8+ T-cell function [129]. In addition to providing insight into the molecular mechanisms underlying metastatic progression, its elevated circulating levels in patients with distant metastatic disease suggest a potential role as a biomarker of metastasis and a candidate for future therapeutic intervention. Similarly, EVs miR-17-5p was enriched in the serum of TC patients with lung metastases. Mechanistic studies on Cal62 cells demonstrated that miR-17-5p promotes aggressive behavior through NF-κB pathway activation, thereby fostering tumor growth and metastatic colonization [88]. Dai et al. found DOCK9-AS2 to be upregulated in both PTC tissues and plasma-derived EVs, while in vitro studies demonstrated CSC-derived EV-associated DOCK9-AS2 to promote proliferation, invasion, EMT, and stemness through activation of the Wnt/β-catenin pathway [99]. These findings implicate DOCK9-AS2 in TC progression and point to its possible utility in patient stratification and targeted treatment approaches. Similarly, EVs miR-655-3p was downregulated in the serum of PTC patients, and experimental studies showed that it inhibits tumor growth, invasion, and M2 macrophage polarization by targeting CXCR4 [90]. The clinical relevance of EV RNAs was further highlighted in studies investigating radioiodine-refractory PTC. EV-associated miR-1296-5p is a promising predictive biomarker of radioiodine-refractory PTC and may contribute to disease pathogenesis through direct suppression of NIS [157]. A tissue-on-chip approach enabled the analysis of sEV-derived miRNAs directly released from thyroid tissues. Differential expression of several miRNAs (miR-375-3p, miR-7-5p, miR-382-5p, and miR-127-3p) was identified in Graves’ disease-derived sEVs, highlighting the utility of tissue-derived EV profiling for biomarker discovery [172].

Therefore, the available evidence indicates that reliance on a single biological source, such as serum, plasma, or cell culture, may provide only a partial and sometimes misleading view of EV-associated biomarkers in TC. Each sample type captures a different biological compartment: cell culture models are useful for mechanistic validation but do not fully reproduce the complexity of the tumor microenvironment; serum and plasma reflect systemic disease-related changes but are influenced by pre-analytical variability, platelet activation, coagulation processes, hemolysis, and the contribution of EVs from non-tumor cells; while tissue, FNA, and tissue-derived EV models may better reflect local tumor biology but are less accessible and may be affected by sampling heterogeneity. Therefore, testing EV cargo across samples of different origin strengthens biomarker interpretation by enabling cross-validation between tumor-derived signals, circulating signatures, and functional experimental models.

From a clinical perspective, multi-source validation is particularly important for distinguishing robust biomarkers from context-dependent molecular changes. Biomarkers detectable in circulation may be more suitable for minimally invasive diagnosis, follow-up, assessment of metastatic risk, and monitoring of radioiodine-refractory disease, whereas concordance with tissue or FNA findings can increase confidence that the signal is tumor-related rather than a nonspecific systemic response. However, several limitations remain, including small and heterogeneous patient cohorts, inconsistent EV isolation and normalization methods, differences between serum and plasma processing, lack of standardized reporting, and limited independent validation. Future studies should therefore integrate matched thyroid tumor tissue, FNA material, serum/plasma EVs, and functional models, using standardized protocols and clinically annotated cohorts, to determine which EV biomarkers are reproducible, biologically meaningful, and applicable in routine TC clinical decision-making.

## 6. Challenges and Future Perspectives

Owing to their stability in biological fluids, protection of molecular cargo, and ability to reflect the molecular characteristics of the tumors from which they originate, EVs have emerged as promising candidates for liquid biopsy, with potential applications in early diagnosis, risk stratification, treatment monitoring, and detection of disease recurrence. At present, however, EV-derived biomarkers are expected to complement—not replace—established TC diagnostic and prognostic methods. They are expected to serve as complementary tools by providing additional insights into tumor biology, thereby enhancing existing molecular and imaging approaches and potentially enabling more personalized clinical decision-making. 

As the field continues to expand rapidly, comprehensive integration of the accumulating evidence becomes increasingly important. In this context, the present review provides a systematic overview of the currently available data on EV cargo in TC while integrating methodological aspects of EV isolation and characterization together with the diversity of biological sample types used across published studies. By combining methodological and biological perspectives within a single framework, this review may facilitate comparison between studies, identify current knowledge gaps, and serve as a useful reference for the design of future experimental, translational, and clinical investigations. 

The successful clinical translation of EV-based technologies into routine practice depends on overcoming several methodological and biological challenges. Standardization of sample collection, EV isolation and characterization protocols, and downstream molecular analyses is essential to improve reproducibility and comparability across studies. The relatively small size of currently available clinical cohorts, crossed with the biological heterogeneity of EVs, continues to hamper biomarker validation. An additional challenge is the lack of validated TC-specific EV markers, making it difficult to distinguish the TC-derived EVs from the large background of vesicles released by healthy tissues. These issues are particularly important because EV profiles may vary across serum, plasma, FNA material, tissue-derived EVs, and cell culture models. Further progress in the field will depend on the integration of advanced technologies, including single-EV analysis, spatial omics, microfluidic platforms, multi-omics approaches and artificial intelligence (AI)-assisted data analysis. Single-EV analysis may improve the resolution of EV heterogeneity, spatial omics may provide insight into the tissue-specific context of EV-mediated communication, while microfluidic technologies may enable rapid and standardized EV isolation from small sample volumes. Multi-omics approaches may improve molecular characterization by integrating transcriptomic, proteomic, lipidomic, and metabolomic EV profiles, and AI-driven analytical tools may facilitate the identification of complex EV biomarker signatures.

Beyond their diagnostic and prognostic value, EVs are also being investigated as therapeutic tools. EVs’ intrinsic biocompatibility, low immunogenicity, and natural cell-to-cell communication properties make them attractive drug delivery vehicles. Engineered EVs or exosome-mimetic nanovesicles could theoretically be used to deliver tumor-suppressive miRNAs, siRNAs, small molecules, or immune-modulatory cargo; restore iodine-handling mechanisms such as NIS expression; inhibit oncogenic pathways including PI3K/AKT, MAPK, NF-κB, Wnt/β-catenin, and CXCR4-related signaling; and enhance sensitivity to radioiodine, kinase inhibitors, or immunotherapy. These possibilities are particularly relevant for aggressive, metastatic, and radioiodine-refractory thyroid disease, where additional therapeutic options are needed.

Nevertheless, despite these encouraging advances, several unresolved issues must be addressed before EV-based therapy can be clinically implemented. A major challenge is the feasibility of commercial-scale EV production. Unlike conventional synthetic nanoparticles, EVs are biologically derived, source-dependent, and intrinsically heterogeneous, meaning that their composition, biological activity, and safety profile may vary according to the parental cell type, culture conditions, isolation method, storage procedure, cargo-loading strategy, and loading efficiency. This raises important questions regarding batch-to-batch reproducibility, purity, potency, stability, sterility, and regulatory classification of EV-based products. Another critical limitation is the absence of standardized dosing principles. At present, it remains unclear whether EV dose should be defined by particle number, total protein content, cargo concentration, biological potency, or a combination of these parameters. This is particularly relevant for therapeutic EVs loaded with miRNAs, siRNAs, drugs, or immune-modulatory molecules, because the same particle dose may not correspond to the same functional dose. In addition, EV pharmacokinetics and biodistribution are strongly influenced by route of administration, vesicle size, surface markers, cellular origin, and uptake by the mononuclear phagocyte system. Accumulation in the liver, spleen, lungs, kidneys, or immune compartments may reduce tumor-specific delivery and increase the possibility of off-target effects. In TC, this is particularly relevant because EVs can have dual, context-dependent roles: while engineered EVs may suppress tumor growth or improve treatment sensitivity, insufficiently characterized EV preparations could theoretically promote tumor progression, angiogenesis, EMT, immune evasion, macrophage polarization, or metastatic niche formation if they carry pro-oncogenic cargo. Thus, therapeutic development must include rigorous source selection, cargo characterization, dose–response assessment, biodistribution analysis, and long-term toxicology studies. 

Overall, EVs represent a highly promising but still emerging field in TC research. Their clinical translation requires harmonized protocols, scalable manufacturing, validated potency assays, clinically meaningful dosing units, long-term safety evaluation, and well-designed trials that monitor tumor response as well as systemic immune, vascular, endocrine, and metabolic effects. Until these issues are resolved, EVs should be viewed as a highly promising but still experimental therapeutic strategy in TC, with a primary clinical relevance in biomarker discovery for minimally invasive disease monitoring and high-risk patient selection.

## Figures and Tables

**Table 2:** Methodological approaches to thyroid cancer extracellular vesicle (EV) characterization.

Method	Principle/How It Works	Application in TC/ Advantages	Limitations
Nanoparticle Tracking Analysis (NTA)	Tracks Brownian motion of particles via laser light scattering and video microscopy in liquid	Quantification of EV size distribution and concentration. Detection of EVs with previously fluorescently labeled surface markers; measures Zeta potential	Limited resolution for very small EVs (<70 nm); operator- and parameter-dependent; can be affected by contaminants
Dynamic Light Scattering (DLS)	Measures fluctuations in scattered light intensity	Determination of particle size distribution in samples; measures Zeta potential	Sensitive to aggregates and polydispersity; less reliable in complex biofluids; good for large particles
Tunable Resistive Pulse Sensing (TRPS)	Measures changes in electrical current as individual vesicles pass through a nanopore	High-precision sizing and concentration measurement; measures Zeta potential	Low throughput; pore clogging; requires careful calibration
Electron Microscopy (EM)	Uses electron beams (TEM/SEM) to visualize particles based on electron scattering/emission	High-resolution imaging; Confirms vesicle morphology and integrity	Labor-intensive; sample prep may introduce artifacts; not quantitative
Western Blot (WB)	Protein separation (SDS-PAGE), membrane transfer, and detection of specific protein markers (e.g., CD63, CD9, TSG101)	Confirms EV identity and protein cargo	Semi-quantitative; low throughput; requires sufficient protein concentrations
Flow Cytometry (FC)	Detects particles individually via light scattering and fluorescence as they pass through a laser beam	Characterization of EV surface markers; population analysis	Limited sensitivity for small EVs; labeling efficiency affects results
Next-Generation Sequencing (NGS)	Massively parallel sequencing of EV-derived nucleic acids following library preparation	Global DNA/RNA profiles (miRNA, lncRNA, mRNA…)	Expensive; raw data complexity; bias toward abundant RNAs; requires sufficient RNA input
Quantitative PCR (qPCR)	Amplifies and quantifies specific nucleic acid sequences	Quantitative measurement of specific DNAs/RNAs	Sensitive to low DNA/RNA yield; primer bias

TEM, transmission electron microscopy; SEM, scanning electron microscopy.

## Data Availability

The data analyzed in this review were obtained from published studies cited throughout the manuscript. The extracted data supporting the conclusions of this review are included in the article and its Supplementary Materials.

## Supplementary Materials

The following supporting information can be downloaded at: https://www.mdpi.com/article/10.3390/ijms27167337/s1. References [173,174,175,176,177,178,179] are cited in the supplementary materials.
