# Supplementary material for: Comprehensive Review of Extracellular Vesicles in Thyroid Cancer: From Methodological Approaches to Biological Functions and Clinical Applications
Source: Int J Mol Sci. 2026 Aug 17;27(16):7337. doi: 10.3390/ijms27167337 (PMC13513305; doi:10.3390/ijms27167337)
Supplement: Supplementary file 1 [file ijms-27-07337-s001.zip › ijms-4451091-supplementary.pdf]

Table S1. Summary of studies on EVs in thyroid cancer identified through a PubMed search using the following strategy: (("Extracellular Vesicles"[MeSH] OR "extracellular vesicles"[Title/Abstract] OR "exosomes"[Title/Abstract] OR "exosome"[Title/Abstract] OR "microvesicle"[Title/Abstract] OR "small extracellular vesicles"[Title/Abstract] OR "microparticles"[Title/Abstract] OR "micro-particles"[Title/Abstract]) AND ("Thyroid Neoplasms"[MeSH] OR "thyroid cancer"[Title/Abstract] OR "thyroid carcinoma"[Title/Abstract])) NOT review[Publication Type] and Goggle search engine using terms: extracellular vesicles, thyroid carcinoma, biomarkers.

The systematic search through a PubMed database (up to 21st april 2026) yielded 113 articles; after screening, 51 were excluded (5 retracted, 6 bioinformatics-focused studies, 1 duplicate, 13 with inaccessible full texts, and 26 unrelated to thyroid-derived EVs or based on book chapters or non-human models). A total of 62 studies were included for analysis of EV isolation methods and molecular cargo characterization. Among these, 12 analyzed serum-derived EVs, 12 plasma-derived EVs, 25 EVs derived from cell lines, 3 urinary EVs, and 10 studies investigated EVs from multiple/other biological sources (e.g., tissue, plasma, and serum). Search through a Goggle search engine yielded 6 additional articles, 1 analyzed serum-derived EVs, 1 plasma-derived EVs and 4 investigated EVs from multiple biological sources.

Ref	EV Source	Isolation Method	Cargo	Key Molecules (with trend)	Application	Key findings
92	Plasma	DUC	miRNA	↑miR-126-3p, ↑miR-145-5p, ↑miR-31-5p, ↑miR-21-5p ↑miR-181a-5p	Diagnosis	miR-31-5p (PTC vs BT)<br>miR-21-5p (PTC vs BT)<br>miR-21-5p, 181a-5p (FTC vs PTC)<br>Plasma EVs miR-21 and miR-181a effectively distinguish FTC from PTC, supporting their role as non-invasive biomarkers for differential diagnosis and improved clinical classification.
93	Plasma	exoRNeasy Serum or Plasma Maxi Kit	miRNA	↑miR-21-5p, ↑miR-146b-5p, ↑miR-204-5p, ↑miR-221-3p, ↑miR-222-3p	LNM prediction/ prognosis	LNM vs non-LNM PTC<br>Plasma EV miR-146b-5p and miR-222-3p are associated with LNM in PTC, supporting their potential as non-invasive biomarkers for predicting metastatic risk and aiding clinical decision-making.
94	Plasma	ExoQuick ™	miRNA	↓let-7f, ↓let-7g, ↓let-7d	Diagnosis	Follicular nodules (FTC vs FTA)<br>miRNA let-7 derived from TPO-positive EVs shows potential as a non-invasive biomarker for differentiating follicular thyroid nodules, supporting its utility in improving diagnostic accuracy.
143	Plasma	UC	Protein	↑TSLP	Immune modulation	TSH stimulation response

144	Plasma	ExoQuick ™	miRNA	↑miR-346, ↑miR-10a-5p, ↑miR-34a-5p	Diagnosis (PTC signature)	PTC vs HC / NG<br>A three-miRNA plasma signature demonstrates strong diagnostic potential for PTC, supporting the use of circulating microRNAs as non-invasive biomarkers for accurate disease detection.
145	Plasma	DUC	miRNA panel	49 miRNAs↑<br>80 miRNAs↓<br>↑miR-5189-3p had most optimal for diagnosis	Diagnosis	PTC vs benign nodules<br>EVs miRNA profiles identified by next-generation sequencing can effectively distinguish malignant from benign thyroid nodules, highlighting their potential as non-invasive diagnostic biomarkers.
146	Plasma (children)	DUC	Protein	↑PD-L1	Prognosis/ Immune suppression	T-cell inhibition<br>EVs PD-L1 correlates with clinical features in pediatric TC and contributes to T-cell suppression, highlighting its role in tumor immune evasion and its potential as both a biomarker and an immunotherapeutic target.
147	Plasma	UC	miRNA panel	↑miR-16-2-3p, ↑miR-223-5p, ↓miR-34c-5p, ↓miR-101-3p, miR-182-5p, miR-223-3p, miR-146b-5p<br>↑miR-16-2-3p, ↑miR-223-5p	Diagnosis	PTC vs NG<br>A panel of plasma EVs miRNAs demonstrates strong potential for differentiating malignant from benign thyroid nodules, supporting their use as non-invasive and accurate diagnostic biomarkers for improving clinical decision-making.
148	Plasma	DUC	miRNA	↑miR-1-3p, ↑miR-206, ↑miR-221-3p	Diagnosis	Early PTC detection<br>EVs-associated miRNAs in plasma show promising potential as non-invasive biomarkers for PTC, supporting their feasibility for clinical application in diagnosis and disease monitoring.
149	Plasma	exoRNeasy Serum/Plasma Maxi Kit	miRNA + protein	↑miR-195-3p, ↓miR-3176, ↓miR-205-5p, ↓mir-208-3p, ↓mir-3529-3p, ↓let-7i-3p; ↑KLK11, ↑A1AG2, ↑SMIM1, ↓CXCL7,↓TBB1,BIP,↓ACTN1	Diagnosis	Malignant vs benign<br>Circulating large EVs analyzed through multi-platform omics approaches show strong potential as diagnostic biomarkers for indeterminate thyroid nodules, improving risk stratification and supporting more accurate clinical decision-making.
150	Plasma	CTB affinity capture of EVs	Protein	↑EPCAM+ sEVs; ↓Claudin-11, ↓Integrin αX, ↓LAG-3	Diagnosis/ Monitoring	Post-operative remission (recurence detection). Single small EV analysis identifies plasma EPCAM+ sEVs as sensitive biomarkers for early diagnosis and postoperative monitoring of TC, supporting their utility in real-time disease detection and follow-up.
151	Plasma	DGUC	miRNA	↑miR-146b-5p, ↑miR-21a-5p	Diagnosis	TC vs TA. Two miRNAs enriched in plasma EV demonstrate potential as

						non-invasive biomarkers for TC, supporting their use in improving diagnostic accuracy and disease detection.
152	Plasma	ExoQuick ™ Exosome Precipitation Solution	miRNA	↑miR-6774-3p, ↑miR-6879-5p	Prognosis/ Metastasis prediction	PTC-N1 vs PTC-N0<br>Plasma EVs miRNAs demonstrate strong potential as non-invasive biomarkers for identifying LNM in PTC, supporting their clinical utility in risk stratification and treatment planning.
70	Serum	DUC, Total Exosome Isolation Reagent	Protein	↑SRC, ↑TLN1, ↑ITGB2, ↑CAPNS1	Diagnosis of LNM/ Prognosis	PTC with LNM<br>Proteomic profiling of serum-derived EVs from PTC patients reveals distinct protein signatures associated with tumor biology, supporting their potential as non-invasive biomarkers for diagnosis and disease characterization.
100	Serum	ExoQuick Exosome Precipitation Solution	circRNA	circRNA_007293	Progression biomarker	EMT/proliferation<br>EVs circRNA hsa_circ_007293 promotes PTC progression by enhancing proliferation, migration, invasion, and EMT through the miR-653-5p/PAX6 axis, highlighting its role as an oncogenic regulator and potential therapeutic target.
101	Serum	exoEasy Maxi Kit	circRNA	multiple circRNAs (684↑ and 2193↓) (*circTACC2*-miR-7-*EGFR* and *circBIRC6*-miR-24-3p- *BCL2L11 axis)*	Prognosis	LNM<br>Serum EVs circular RNA profiles differ in PTC patients with LNM, highlighting their potential as non-invasive biomarkers for metastatic risk assessment and disease progression.
102	Serum	UC	circRNA	↑hsa_circRNA_007293, ↑hsa_circRNA_031752, ↑hsa_circRNA_020135	Diagnosis	PTC progression<br>Altered circular RNA profiles in serum-derived EVs from PTC patients reveal distinct expression patterns, highlighting their potential as novel non-invasive biomarkers for disease detection and characterization.
116	Serum	DUC	miRNA	↑miR-423-5p	Diagnosis/ Prognosis/ Therapy	EVs miR-423-5p promotes oncogenic activity in PTC, supporting its role as both a diagnostic biomarker and a potential therapeutic target.
118	Serum	UC, phosphatidylserine	Protein	↑RAB21	Diagnosis	FTC vs FTA<br>RAB21 is elevated in serum-derived EVs from patients with FTC, highlighting its potential as a non-invasive proteomic biomarker for

		affinity method				disease detection and characterization.
153	Serum	DUC (microvesicles isolation)	Microparticles	↑procoagulant MPs	Monitoring	TSH stimulation
154	Serum	ExoQuick Exosome Precipitation Solution	miRNA	↑miR-25-3p, ↑miR-296-5p, ↑miR-92a-3p	Diagnosis	PTC<br>A three-miRNA serum panel demonstrates strong diagnostic performance for PTC, supporting its potential as a reliable and non-invasive biomarker for disease detection.
155	Serum	ExoQuick Exosome Precipitation Solution	miRNA	↓miR-29a	Diagnosis/ Prognosis	PTC vs control<br>Decreased serum EVs miR-29a expression is associated with PTC and may serve as a non-invasive biomarker with clinical relevance for diagnosis and disease assessment.
156	Serum	ExoQuick Exosome Precipitation Solution	miRNA	↑miR-24-3p, ↑miR-146a-5p, ↑miR-181a-5p, ↑miR-382-5p	Diagnosis	PTC and healthy controls<br>Serum EVs microRNA profiles differ between PTC patients and controls, supporting their potential as non-invasive biomarkers for disease detection and characterization.
157	Serum	SEC (qEV IZON™)	Protein	↑BST2	Diagnosis/ Prognosis/ Therapy	Micro PTC vs control (benign thyroid nodule)<br>Serum small Ev-derived BST2 is elevated in papillary thyroid microcarcinoma and promotes LNM, highlighting its dual role as a functional mediator of tumor progression and a promising non-invasive biomarker for metastatic risk.
158	Serum	DUC	Protein	↑TACSTD2	Diagnosis	Tumor susceptibility. Single small EV profiling identifies TACSTD2^{+} sEVs as a population associated with increased tumor susceptibility in elderly individuals, highlighting their potential as biomarkers for cancer risk and early detection.
159	Serum	DUC, ExoQuick	miRNA	↑miR-222-3p	Diagnosis/ Monitoring/	PTC<br>An enhanced electrochemiluminescence platform based on MBene

		Exosome Precipitation Solution			Therapy	quantum dots with a nanoparticle-on-mirror structure enables highly sensitive detection of EV miRNAs, supporting its potential as an advanced analytical tool for early diagnosis and biomarker detection.
160	Urine	UC, ultrafiltration, ExoQuick-TC	Peptides	↑Tg, ↑Galectin-3, ↑ANXA2, ↑ANGPT1, AFM, S100A8, S100A9 peptides, TIMP, Keratin-8, Keratin-19	Monitoring	Prediction of reccurence<br>Post-thyroidectomy<br>Urinary EV peptide profiles undergo significant long-term changes following thyroidectomy in TC patients, potential as non-invasive biomarkers for postoperative monitoring and disease follow-up.
161	Urine	UC, ultrafiltration, ExoQuick-TC	Protein	↑TIMP, ↑ANGPT1	Diagnosis	Early detection of differentiated TC using urine EV proteins<br>Urinary EV TIMP and ANGPT1 are elevated in WDTC and show potential as novel non-invasive preoperative biomarkers for disease detection.
162	Urine	UC, ultrafiltration, ExoQuick-TC	Protein	↑Tg, Galectin-3	Monitoring/ Reccurence	Post-ablation<br>Urinary EV thyroglobulin represents a novel non-invasive biomarker for monitoring TC patients after ablative therapy, with potential utility in disease surveillance and follow-up.
69	PTC and ATC cell lines	DUC	Protein	multiple	Diagnosis/ Prognosis/ Therapy	EVs derived from TC cells carry functional components that actively participate in ECM remodeling, thereby promoting tumor invasion and progression and highlighting their role in shaping the tumor microenvironment.
82	PTC cell lines	DUC	miRNA	Multiple (Global RNA profiling (~20,000 genes) ↑miR-21-5p, ↑miR-31-5p, ↑miR-221-3p, ↑miR-222-3p, ↑let-7i-3p, ↑miR-24-3p, ↑miR-31-3p, and ↑miR-376a-3p	Diagonsis/ Prognosis/ Therapy	EVs microRNAs derived from PTC cells regulate multiple target genes involved in tumor progression, highlighting their role in intercellular communication and their potential as biomarkers and therapeutic targets.
83	PTC, non-tumor cell lines	ExoQuick-TC	RNA profile	n/A	Diagnosis/ Prognosis/ Therapy	Tumorigenic and non-tumorigenic thyroid cells exhibit distinct RNA profiles both intracellularly and within their released EV, indicating selective RNA packaging and highlighting the role of EV RNA in tumor-

						related communication and as a potential source of biomarkers.
85	cell lines TPC and NTHY	DUC	miRNA	↑miR-146b, ↑miR-222	Mechanistic insight/ Diagnosis (PTC and recidives)	PTC-derived exosomes are enriched with miR-146b and miR-222, supporting their role as tumor-associated extracellular miRNAs and highlighting their potential as non-invasive biomarkers for thyroid cancer detection and monitoring.
86	hypoxic PTC cells	DUC	miRNA Protein	↑miR-181a,↓MLL3, ↓DACT2 ↑YAP, ↑VEGF	Signaling pathways/ Prognosis/ Therapy/ Diagnosis	Hypoxia-induced EV miR-181a promotes angiogenesis in PTC by suppressing DACT2 via MLL3 downregulation and activating the YAP–VEGF pathway, highlighting its role as an oncogenic mediator of tumor vascularization and a potential therapeutic target.
88	B-CPAP TPC-1 cell lines	DUC (max 10,000 x g)	miRNA	↑miR-152	Therapeutic targeting/ Prognosis	Mesenchymal stem cell–derived EVs miR-152 suppresses TC progression by targeting DPP4, leading to reduced proliferation, invasion, and migration, and highlighting its potential as a therapeutic agent in TC.
91	ATC cell line	UC	miRNA	↑miR-210-3p	Mechanistic insight	miR-210 is strongly induced by hypoxia in ATC and is actively released via EV and Argonaute-2 complexes, highlighting its role as a hypoxia-responsive biomarker and a potential mediator of tumor microenvironment signaling.
95	PTC cell line and Nthy-ori-3	UC, PEG precipitation	lncRNA Protein	↑SNHG9 (lncRNA) YBOX3 ↑P21 (CDKN1A) ↓Autophagy ↑Apoptosis	Tumor biology/ Diagnosis/ Prognosis/ Therapy	↓ autophagy, ↑ apoptosis<br>EV-enriched lncRNA SNHG9 derived from PTC cells alters neighboring normal thyroid epithelial cells by inhibiting autophagy and promoting apoptosis via the YBOX3/P21 pathway, highlighting a mechanism of tumor–normal cell interaction mediated by extracellular vesicles.
96	ATC cell line	DUC	lncRNA miRNA Protein	↑FGD5-AS1 (lncRNA) ↓miR-6838-5p ↑VAV2 Angiogenesis	Diagnosis/ Prognosis/ Therapy	Tumor-derived EVs lncRNA FGD5-AS1 promotes angiogenesis, vascular permeability, and metastasis in thyroid cancer by regulating the miR-6838-5p/VAV2 axis, highlighting its role as an oncogenic mediator and potential therapeutic target.
97	PTC, ATC and FTC cell lines	UC, Precipitation	lncRNA miRNA Protein	↑CDKN2B-AS1 (lncRNA) ↓miR-122-5p ↑P4HA1	Diagnosis/ Prognosis/ Therapy	EVs lncRNA CDKN2B-AS1 derived from cancer stem cell-like cells promotes TC progression by regulating the miR-122-5p/P4HA1 axis, enhancing proliferation, migration, and invasion, and highlighting its role as an oncogenic mediator and potential therapeutic target.
99	CSCs and	ExoQuick	lncRNA	↑lncRNA	Prognostic	EVs derived from thyroid cancer stem-like cells promote EMT through

	PTC cell lines	TC			markers/ Targeted therapy	the transfer of lncRNAs, thereby enhancing tumor invasiveness and highlighting a key mechanism of intercellular communication driving TC progression.
103	ATC cell line	UC	siRNA	SCD-1 siRNA	Therapy (drug delivery system)	↑ ROS, ↑ cell death<br>EV-mediated delivery of SCD-1 siRNA induces cell death in ATC by increasing ROS levels, demonstrating a promising therapeutic strategy that combines targeted gene silencing with EV–based delivery systems.
112	PTC and ATC, fibroblasts cell lines	UC	Protein	CD147	Therapeutic relevance	EV mediate bidirectional communication between thyroid tumor cells and fibroblasts, promoting a tumor-supportive microenvironment and enhancing cancer progression, highlighting their role in stromal–tumor interactions and potential as therapeutic targets.
139	BCPAP and Nthy-ori-3-1 cell lines	DUC	Protein	Multiple ↑miR-21-5p (targeting TGFBI and COL4A1)	Diagnosis/ Prognosis/ Therapy	EVs derived from PTC cells enhance angiogenesis within the tumor microenvironment, promoting tumor progression and highlighting their role as key mediators of pro-angiogenic signaling and potential therapeutic targets.
163	PTC, FTC, ATC cell line	ExoQuick-TCTM	mRNA, protein, EV number	Tetraspanins CD9, CD63, CD81, CD151 level of gene expression	Mechanistic insight	Total extracellular vesicles derived from TC cells do not alter proliferation, viability, migration, or invasion of normal thyroid cells but modulate tetraspanin expression and suppress angiogenesis, with larger (~6 μm) vesicle fractions potentially exerting distinct cell-specific effects.
164	Cell lines (ATC)	Centrifugation, precipitation	Transcriptomics, proteomics	N/A	Therapy prediction	EV-associated transcriptomic and proteomic biomarkers demonstrate strong in vitro potential for monitoring response to vandetanib treatment in ATC, supporting their use as non-invasive tools for therapy monitoring and personalized treatment optimization.
165	PTC cell line	DUC	Not reported	MAPK signaling pathway ERK phosphorylation	Treatment monitoring	Inhibition of the MAPK pathway alters EV release dynamics, indicating that EV secretion is a regulated cellular response to targeted therapy and may reflect adaptive tumor signaling mechanisms, with potential implications for treatment monitoring and resistance.
166	ATC and FTC cell lines	DUC	Protein	TSHR	Therapeutic relevance	EVs expressing the thyrotropin receptor (TSHR) can bind and neutralize stimulating autoantibodies, thereby attenuating cAMP production, suggesting a novel regulatory mechanism and potential therapeutic approach in autoimmune thyroid diseases
167	ATC and	DUC	Protein	HSP-related	Therapeutic	The heat shock protein inhibitor KU757 effectively suppresses growth of

	FTC cell lines				relevance/ Prognosis	lenvatinib-resistant FTC cells by targeting metabolic reprogramming, particularly glycolysis, highlighting its potential as a therapeutic strategy to overcome drug resistance.
168	Cal62 and NK92-MI cell lines	DUC (omitted 10,000 x g step)	Not reported	radioiodine labeling of EVs	Diagnosis/ Therapy	Radioiodine labeling enables effective in vivo tracking of EVs, providing a valuable tool for studying their biodistribution and supporting their potential use in diagnostic imaging and targeted therapeutic delivery.
169	FTC cell line	Centrifugation (ExoView analysis)	Protein	CD63, CD81, CD9	Tumor biology	Prolonged exposure to real microgravity alters EV release and composition in TC cells, indicating that environmental conditions can significantly influence EV–mediated communication and tumor cell behavior.
170	thyroid cell line SW579	UC	Protein	↑ANXA1	Diagnosis/ Prognosis/ Therapy	EVs ANXA1 derived from TC cells promotes malignant transformation of normal thyroid follicular epithelial cells by enhancing proliferation, highlighting its role in tumor initiation and intercellular communication within the tumor microenvironment.
171	FTC, ATC, non-tumor cell lines	DUC (microvesicles isolation)	Protein	many	Diagnosis/ Prognosis/ Therapy	Comparative proteomic analysis of ectosomes from TC and normal thyroid cells reveals distinct protein signatures associated with cancer-related processes, highlighting their potential functional roles in tumor progression and their value as candidate biomarkers.
172	FTC and ATC cell lines	DUC	Protein	Multiple	Diagnosis/ Prognosis/ Therapy	Proliferation ↑, migration ↑, invasion ↑<br>Proteomic profiling of thyroid carcinoma-derived EVs reveals a diverse set of proteins involved in carcinogenesis and thyroid homeostasis, highlighting their functional roles in tumor progression and their potential as biomarkers and therapeutic targets.
173	ATC, non-tumor cell lines	DUC	Engineered therapeutic cargo	TMTP1 (targeting peptide)<br>CRISPR/Cas9<br>BRAF mutation<br>Vemurafenib	Therapy/ Overcoming vemurafenib resistance	Innovative combination of gene editing technology with a nanoparticle delivery system shows promising potential as a therapeutic strategy for treating aggressive BRAF-mutant ATC.
61	Nthy-Ori 3-1, TPC-1, OCUT2 cell lines/plasm	DUC, SEC	Protein	TSHR, Tg	Targeted isolation/ Prognosis	Thyroid-derived EVs retain TSHR on surface and carry Tg as cargo, with vesicular Tg detectable in recurrent thyroid cancer even in Tg-Ab–positive patients, supporting TSHR-based EV enrichment and proposing EV-associated Tg as a potential recurrence biomarker when serum Tg is

	a					unreliable.
72	Plasma, tissue	DUC, UF	Protein	↑Hsp27, ↑Hsp60, ↑Hsp70, and ↑Hsp90	Diagnosis	Molecular chaperones exhibit distinct expression patterns in thyroid tissues and circulating EVs under normal and pathological conditions, highlighting their potential as clinically relevant biomarkers and their involvement in thyroid disease pathophysiology.
87	Serum, cell line (Cal62)/Tissue	DUC	miRNA	↑miR-17-5p<br>↑NF-κB signaling pathway<br>IL-6<br>IL-8	Diagnosis/ Prognosis/ Therapy	EVS miR-17 functions as an oncogenic driver of thyroid cancer metastasisthrough activation of the NF-κB pathways
89	PTC cell lines, tissue	DUC	miRNA<br>Protein	↓miR-655-3p<br>↓CXCR4<br>↓Ki67<br>↓CD163	Treatment strategy/ Diagnosis/ Prognosis	EVs miR-655-3p inhibited growth, invasion and macrophage M2 polarization through targeting CXCR4 in PTC
90	Tissue, cell lines	Total Exosomes Isolation Kit	miRNA<br>Protein	↓miR-145<br>↓AKT3	Diagnosis/ Prognosis/ Therapy	Reduced miR-145 expression may serve as a potential biomarker for thyroid cancer. Low levels of miR-145 may be associated with more aggressive disease and metastatic potential.miR-145 restoration could represent a novel therapeutic strategy
98	Plasma, cell lines	Centrifugation, Exoquick exosome precipitation	lncRNA	↑DOCK9-AS2<br>↑Wnt/β-catenin signaling pathway	Diagnosis/ Prognosis/ Therapy	EVs lncRNA DOCK9-AS2 derived from cancer stem cell-like cells promotes PTC progression by activating the Wnt/β-catenin pathway
111	Plasma, tissue, cell lines (PTC)	DUC, ExoQuick Exosome Precipitation Solution	miRNA	↑miR-221-3p	Diagnosis/ Prognosis	EVs derived from hypoxic PTC cells transfer miR-221-3p to normoxic tumor cells, promoting tumor progression through regulation of ZFAND5 and highlighting a hypoxia-driven mechanism of intercellular communication.
130	Tissue, TC cell lines	DUC	miRNA	↑miR-519e-5p	Diagnosis/ Prognosis/ Therapy	EVs miR-519e-5p acts as an oncogenic regulator in metastatic PTC, promoting tumor progression and immune escape by inducing $CD8^{+}$ T-cell exhaustion

134	Dental pulp EVs, PTC cell lines	Microfiltration, UC	Protein miRNA	N/A	Diagnosis/ Prognosis/ Therapy	EV derived from human immature dental pulp cells have anti-tumor effects in PTC, supporting their potential use as a novel therapeutic approach
174	Tissue, serum, cell line	Total Exosomes Isolation Kit	miRNA	↓miR-1284 tissue ↑miR-1284 serum	Diagnosis/ Prognosis/ Therapy	miR-1284 functions as a tumor suppressor in TC, primarily by inhibiting proliferation and migration of cancer cells
175	TC cell lines,tissue	DUC	miRNA	↑miR-375-3p ↑ miR-7-5p ↑miR-382-5p ↑miR-127-3p	Diagnosis/ Prognosis/ Therapy	Tissue-on-chip technology is a powerful tool for improving the discovery of EVs miRNA biomarkers
177	Plasma, Cell Lines, tissue	UC	miRNA	↑ miR-1296-5p	Prognosis/ Therapy (RAIR PTC)	RAIR vs non-RAIR<br>Prediction of resistency to radioiodine thrapy, Prediction of outcome, Potential therapy<br>Circulating EvS miRNAs are associated with radioiodine-refractory PTC and may serve as non-invasive biomarkers for predicting treatment resistance and guiding personalized therapeutic strategies.
178	Plasma, tissue	UC	miRNA	miRNA panel; ↑miR-127-3p, ↑miR-223-5p, ↑miR-432-5p, ↑miR-146a-5p, ↑miR-151a-3p	Diagnosis/ Prognosis	FTC vs FA<br>A circulating small extracellular vesicle (sEV)-based miRNA classifier demonstrates high diagnostic accuracy for FTC, supporting its potential as a non-invasive tool for precise disease detection and improved clinical classification.
179	Plasma, Serum	DUC	miRNA	↑miR-485-3p, ↑miR-4433a-5p, ↑miR-4306, ↑miR-376a-3p, ↑miR-204-3p	Diagnosis/ Prognosis	TC vs TA<br>EVs miRNAs identified through small RNA sequencing show strong potential as non-invasive diagnostic biomarkers for PTC, supporting their role in improving early detection and clinical diagnosis.

ACTN1 - actinin alpha 1

AFM - afamin

AKT3 - AKT serine/threonine kinase 3

ANGPT1 - angiopoietin-1

ANXA1 - annexin A1

ANXA2 - annexin A2

ATC - anaplastic thyroid carcinoma

BCL2L11 - BCL2-like protein 11 (BIM)

BiP - binding immunoglobulin protein

BST2 - bone marrow stromal antigen 2

CAPNS1 - calpain small subunit 1

CD - cluster of differentiation

circRNA - circular RNA

CSCs - cancer stem-like cells

CTB - cholera toxin subunit B

CXCL7 - C-X-C motif chemokine ligand 7

CXCR4 - C-X-C chemokine receptor 4

DOCK9-AS2 - DOCK9 antisense RNA 2

DUC – differential ultracentrifugation

ECM - extracellular matrix

EGFR - epidermal growth factor receptor

EPCAM - epithelial cell adhesion molecule

EVs - extracellular vesicles

ExoQuick / ExoQuick-TC / Exosome Precipitation Solution / Total Exosome Isolation Kit / Total Exosome Isolation Reagent / ExoQuick Exosome Precipitation Solution – polymer-based precipitation

exoEasy Maxi Kit / exoEasy Midi Kit / exoRNeasy - membrane affinity capture + RNA extraction

FGD5-AS1 - FYVE, RhoGEF and PH domain containing 5 antisense RNA 1

FTC - follicular thyroid cancer

Hsp - heat shock protein

ITGB2 - integrin beta 2

Ki-67 - marker of cellular proliferation

KLK11 - kallikrein-related peptidase 11

lncRNA - long non-coding RNA

LNM - lymph node metastasis

MAPK - mitogen-activated protein kinase

miRNA (miR) - microRNA

MLL3 - myeloid/lymphoid or mixed-lineage leukemia protein 3 (KMT2C)

NK - natural killer

P4HA1 - prolyl 4-hydroxylase subunit alpha 1

PD-L1 - programmed death-ligand 1

PEG - polyethylene glycol

PMPs - platelet-derived microparticles

PTC - papillary thyroid cancer

RAB21 - Ras-related protein Rab-21

S100A8 - S100 calcium-binding protein A8

S100A9 - S100 calcium-binding protein A9

SEC - size exclusion chromatography

SMIM1 - small integral membrane protein 1

SNHG9 - small nucleolar RNA host gene 9

SRC - SRC proto-oncogene, non-receptor tyrosine kinase

TBB1 - tubulin beta-1 chain

TC - thyroid cancer

Tg - thyroglobulin

TIMP - tissue inhibitor of metalloproteinases

TLN1 - talin-1

TSH - thyroid-stimulating hormone

TSHR - thyroid-stimulating hormone receptor

TSLP - thymic stromal lymphopoietin

UC - ultracentrifugation

UF - ultrafiltration
